# Melatonin suppresses ILC2-driven airway hyperreactivity via glutathione-dependent metabolic reprogramming

**DOI:** 10.3389/fimmu.2026.1845654

**Published:** 2026-05-11

**Authors:** Jafar Cain, Benjamin P. Hurrell, Stephen Shen, Paul Speliakos, Omid Akbari

**Affiliations:** Department of Immunology and Immune Therapeutics, Keck School of Medicine, University of Southern California, Los Angeles, CA, United States

**Keywords:** allergic asthma, immunometabolism, innate immunity, innate lymphoid Cells (ILC2), pentose phosphate pathway, redox homeostasis and signaling, respiratory immunology

## Abstract

Allergic asthma is characterized by type 2 inflammation and overnight worsening of symptoms, yet dynamic fluxes in cellular metabolic profiles driving time-of-day variation remain poorly defined. Group 2 innate lymphoid cells (ILC2s) are central mediators of airway hyperreactivity. We identify melatonin as a previously unrecognized regulator of ILC2 metabolism and function. In murine models of allergic airway inflammation, melatonin reduced eosinophilia, type 2 cytokine production, and airway hyperreactivity without altering ILC2 abundance. Mechanistically, melatonin acted independently of canonical melatonin receptors and instead reprogrammed ILC2 metabolism toward pentose phosphate pathway activity, enhancing NADPH generation and NRF2-dependent glutathione accumulation. Metabolic profiling, loss-of-function approaches, and pharmacologic activation studies demonstrated that NRF2 is both necessary and sufficient to restrain ILC2 effector function. Importantly, primary human ILC2s exhibited conserved NRF2 activation, glutathione accumulation, and reduced type 2 cytokine production in response to melatonin, underscoring clinical relevance. Together, these findings identify the melatonin-NRF2-glutathione axis as a metabolic checkpoint regulating innate type 2 immunity and suggest that therapeutic targeting of redox metabolism may represent a strategy for modulating airway inflammation in allergic asthma.

## Introduction

Asthma, a chronic inflammatory disease of the lung, affects up to 300 million people worldwide and remains a major cause of morbidity and mortality (1, 2). In the majority of patients, disease is characterized by type 2 inflammation, featuring eosinophilia infiltration of the airways, mucus production and airway hyperreactivity, manifesting in symptoms such as shortness of breath, wheeze, and cough (3, 4). While adaptive Th2 cells have long been implicated in asthma pathogenesis, group 2 innate lymphoid cells (ILC2s) are now recognized as critical initiators and amplifiers of allergic airway inflammation. ILC2s respond rapidly to epithelial-derived alarmins such as IL-33, IL-25, and thymic stromal lymphopoietin, producing large quantities of IL-5 and IL-13 independently of adaptive immune system (5, 6). Importantly, pulmonary ILC2s exhibit considerable functional plasticity, and their activity is highly sensitive to local tissue-derived signals, positioning them as key regulators of airway inflammation and hyperreactivity.

Despite advances in understanding the cellular drivers of asthma, several fundamental features of the disease remain poorly explained. One of the most striking is the pronounced circadian variation in symptom severity. Asthma symptoms characteristically worsen overnight, with peak airway obstruction and hyperreactivity occurring in the early hours of the morning (7–9). Aurelianus, a Roman physician writing back in the second century, noted that the disease the Greeks were calling ‘Asthma’ affected patients “*atque nocte magis, quam die*” [more at night than in the day] (10). Clinically, nocturnal worsening of asthma is associated with increased risk of severe exacerbations and asthma-related mortality, which peak around 04:00 (8, 9). Moreover, disruption of circadian rhythms, most notably through night-shift work, is strongly associated with increased asthma prevalence and disease severity (11–14). Together, these observations suggest that circadian-regulated physiological signals play a direct and clinically meaningful role in shaping airway inflammation, yet the immune mechanisms underlying this temporal susceptibility remain incompletely understood.

Among circadian-regulated hormones, melatonin is of particular interest. Secreted predominantly by the pineal gland, melatonin coordinates circadian rhythms and peaks during the night in healthy individuals. In patients with asthma, melatonin secretion is frequently dysregulated and has been associated with nocturnal symptom worsening (15). Our findings support prior clinical observations that melatonin signaling is dysregulated in asthma and highlight the importance of temporal and cell-intrinsic context in determining its immunomodulatory effects. In patients with asthma, nocturnal melatonin secretion is often phase-shifted relative to healthy individuals, which may limit the ability of immune cells to engage melatonin-dependent regulatory programs at an appropriate time (15). Our data demonstrate that in ILC2s, melatonin suppresses effector function through a receptor-independent metabolic mechanism involving NRF2 activation and glutathione biosynthesis. Consistent with this, pharmacologic activation of NRF2 phenocopied the effects of melatonin, indicating that engagement of this pathway is sufficient to restrain ILC2 activity independently of melatonin itself. Together, these findings suggest that circadian susceptibility in asthma may reflect impaired temporal coordination and intracellular competence of innate immune cells to execute melatonin-dependent redox buffering programs and identify the NRF2 glutathione axis as a therapeutically actionable pathway.

Beyond its chronobiological role, melatonin exerts pleiotropic effects on immune and respiratory function (16, 17). In murine models of allergic airway disease, exogenous melatonin administration has been reported to reduce pulmonary eosinophilia and type 2 cytokine production (18). However, the immune cell-intrinsic mechanisms by which melatonin influences airway inflammation, and whether it directly regulates ILC2 effector function, remain undefined.

A well-established but underexplored function of melatonin is its role in regulating cellular redox homeostasis. Melatonin enhances the synthesis and recycling of glutathione (GSH), a major intracellular antioxidant critical for controlling redox-sensitive signaling pathways (19, 20). Perturbations in antioxidant defenses are increasingly recognized as key contributors to allergic airway disease, and patients with asthma frequently exhibit reduced glutathione levels in the airway (21). Glutathione synthesis and maintenance require coordinated metabolic inputs, including NADPH generated through the pentose phosphate pathway, and are transcriptionally regulated by nuclear factor erythroid 2-related factor 2 (NRF2) (22–24). NRF2 activity is constrained by its cytoplasmic repressor KEAP1, and in silico and biochemical studies suggest that melatonin may disrupt KEAP1-NRF2 interactions, promoting NRF2-dependent transcription of antioxidant genes such as *Gclc* (25). Additionally, glutathione biosynthesis depends on cysteine availability, which is regulated by transporters such as System Xc^-^, a pathway also reported to be enhanced by melatonin in airway epithelial cells (25).

In this study, we investigated the role of melatonin in regulating ILC2-dependent allergic airway inflammation. We identify a previously unrecognized metabolic reprogramming of ILC2s induced by melatonin, characterized by diversion of glucose and mitochondrial metabolism toward glutathione synthesis. We demonstrate that melatonin-driven glutathione accumulation suppresses ILC2 effector cytokine production and ameliorates airway hyperreactivity and inflammation in multiple murine models of allergic airway disease. Mechanistically, we show that this effect occurs independently of canonical melatonin receptor signaling in ILC2s and instead requires activation of the NRF2-glutathione axis. Finally, we establish that this pathway is conserved in human ILC2s and can be therapeutically manipulated using clinically relevant NRF2-modulating agents. Together, our findings reveal a direct link between melatonin signaling, immune cell metabolism, and type 2 airway inflammation, and identify the melatonin-NRF2-glutathione axis as a potential therapeutic target in asthma.

## Results

### Administration of exogenous melatonin alleviates allergic airways disease severity in allergen-exposed mice

**Figure 1:**
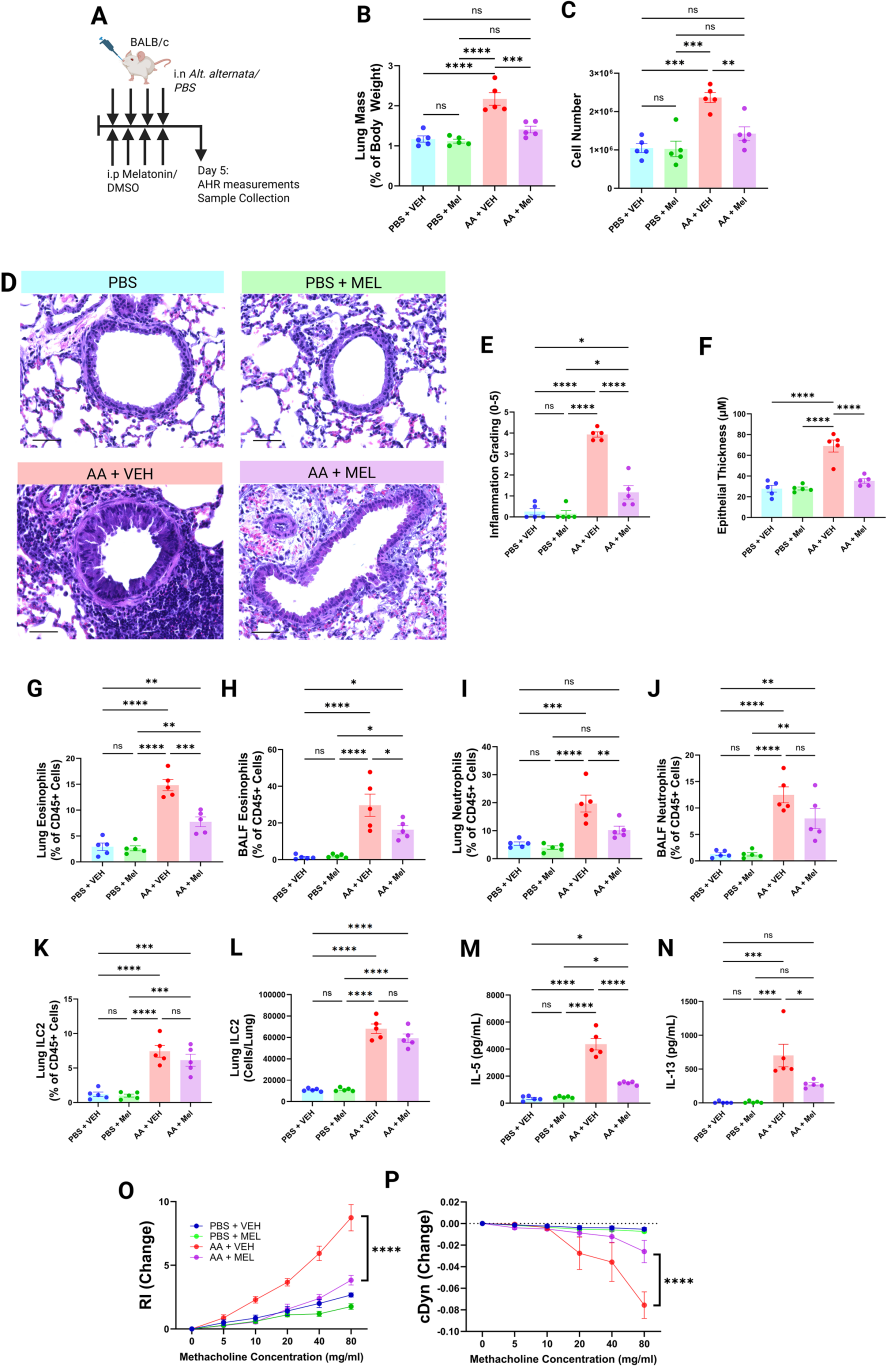
Administration of exogenous melatonin alleviates allergic airways disease severity in allergen-exposed mice. **(A)** Schematic of the *Alternaria alternata* challenge protocol. BALB/c mice were intranasally challenged with 25 μg Alternaria or PBS for four consecutive days, with or without concurrent melatonin, prior to airway hyperreactivity (AHR) measurements and tissue collection on Day 5. **(B)** Lung mass-to-body weight ratios. **(C)** Total BALF cell counts. **(D)** Representative H&E-stained lung sections (40× magnification; scale bar = 100 μm). **(E)** Inflammation scores from H&E-stained FFPE lung sections (mean of three independent blinded scorers). **(F)** Airway epithelial thickness (eight measurements per airway, ten airways per mouse, analyzed in QuPath). **(G–K)** Lung eosinophils **(G)**, BALF eosinophils **(H)**, lung neutrophils **(I)**, BALF neutrophils **(J)**, and lung ILC2s **(K)** quantified by flow cytometry as a proportion of total CD45^+^ cells. **(L, M)** BALF IL-5 **(L)** and IL-13 **(M)** concentrations. **(N, O)** Airway resistance **(N)** and dynamic compliance **(O)** in response to methacholine. ns = P>0.05, *P< 0.05, **P< 0.01, ***P< 0.001, ****P< 0.0001.

Given the growing evidence linking circadian disruption to increased severity of allergic asthma (9, 12, 14, 26, 27), we investigated whether exogenous melatonin could modify disease outcomes in a murine model of allergic airways inflammation. To this end, BALB/c mice, which are genetically deficient in endogenous melatonin production (28), were intranasally challenged with 25 µg *Alternaria alternata* (AA) or PBS daily for four days, with or without concurrent intraperitoneal melatonin treatment using established protocols described previously (18, 29–31) (**Figure 1A**). We then assessed multiple hallmarks of allergic airways disease across a range of physiological, histological, and immunological parameters.

Gross pathological changes expected in this disease model included increased lung mass, increased cellularity of the bronchoalveolar lavage fluid (BALF), epithelial thickening, and pockets of immune cell infiltrations in the lung parenchyma. We noted that the lung to body weight ratio was indeed elevated in AA-exposed mice compared to PBS and melatonin-only controls, however the extent of this increase was substantially reduced in those mice which received melatonin alongside AA (**Figure 1B**). Likewise, the overall cellularity of the BALF sampled from AA-exposed mice was increased compared to PBS and melatonin-only controls (**Figure 1C**). This increase was significantly lower however in melatonin-treated AA-exposed mice (**Figure 1C**). Histological analysis confirmed marked peribronchial and parenchymal immune infiltration following AA exposure, along with significant epithelial thickening - both of which were markedly reduced by melatonin treatment (**Figures 1D-F**).

Closer immunophenotyping of the cellular content of the BALF and lung tissue by flow cytometry showed that AA-exposed mice had significantly greater levels of eosinophilia in both compartments compared to PBS and melatonin-only controls (**Figures 1G, H**). This eosinophilia, in both the lung and BALF, was discernibly lower in mice treated with melatonin alongside AA-exposure (**Figures 1G, H**). Eosinophils are of particular interest in models of allergic airways disease since these are the major effector leukocyte driving pathology (32–36). AA-exposure also precipitated an increase in pulmonary neutrophilia, which is expected in these models (37), which melatonin treatment then also abated (**Figures 1I, J**). While melatonin significantly reduced pulmonary eosinophil and neutrophil numbers, it did not significantly alter the size of the ILC2 compartment in either relative or absolute terms (**Figures 1K, L**). Despite unchanged ILC2 numbers, melatonin significantly reduced BALF IL-5 and IL-13 levels (**Figures 1M, N**), suggesting functional suppression of type 2 cytokine production rather than depletion of ILC2s (**Figures 1K, L**).

Airway hyperreactivity, a cardinal feature of allergic airways disease, was also measured. We found that AA-exposed mice exhibited significantly greater airway hyperreactivity compared to PBS and melatonin-only controls (**Figure 1O**), alongside reduced dynamic compliance (**Figure 1P**). However, in keeping with our other observations, melatonin-treated AA-challenged mice exhibited significantly improved airway function, with reduced hyperreactivity and restored dynamic compliance (**Figures 1O, P**).

### Melatonin exerts protective effects in an ILC2-biased model of allergic airways disease, even in the presence of canonical receptor blockade

**Figure 2:**
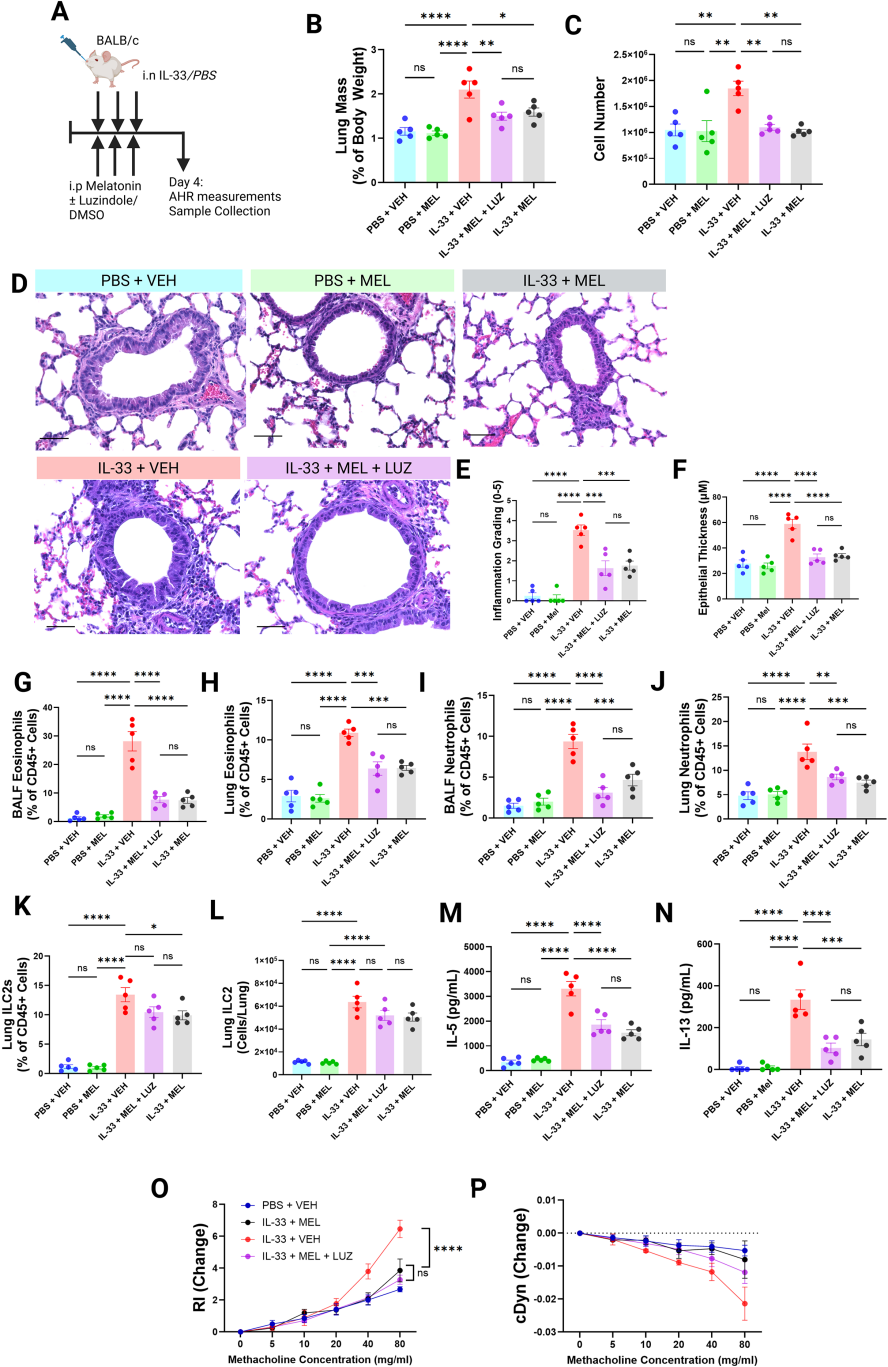
Melatonin exerts protective effects in an ILC2-biased model of allergic airways disease, even in the presence of canonical receptor blockade. **(A)** Schematic of the IL-33 challenge protocol with or without melatonin (MEL) and Luzindole (LUZ), with AHR measurements and tissue collection on Day 4. **(B)** Lung mass-to-body weight ratios. **(C)** Total BALF cell counts. **(D)** Representative H&E-stained lung sections (40× magnification; scale bar = 100 μm). **(E)** Inflammation scores from H&E-stained FFPE lung sections (mean of three independent blinded scorers). **(F)** Airway epithelial thickness (eight measurements per airway, ten airways per mouse, analyzed in QuPath). **(G–K)** Lung eosinophils **(G)**, BALF eosinophils **(H)**, lung neutrophils **(I)**, BALF neutrophils **(J)**, and lung ILC2s **(K)** quantified by flow cytometry as a proportion of total CD45^+^ cells. **(L)** total lung ILC2s quantified by flow cytometry. **(M, N)** BALF IL-5 **(M)** and IL-13 **(N)** concentrations. **(O, P)** Airway resistance **(O)** and dynamic compliance **(P)** in response to methacholine. Data are mean ± SEM; n = 5 per group. One-way ANOVA was used for single-endpoint comparisons; methacholine dose–response curves were analyzed by two-way ANOVA with Tukey’s multiple comparisons. ns = P>0.05, *P< 0.05, **P< 0.01, ***P< 0.001, ****P< 0.0001.

To determine whether the protective effects of melatonin extended beyond allergen-driven disease, we next employed an ILC2-biased IL-33 model of allergic airways inflammation (**Figure 2A**). The IL-33 model was specifically selected to isolate ILC2-intrinsic responses independent of upstream epithelial-derived alarmin complexity present in the previously employed Alternaria model (**Figure 1**). BALB/c mice were intranasally administered 0.5 µg recombinant murine IL-33 while concurrently receiving intraperitoneal injections of melatonin (10 mg/kg) and Luzindole (30 mg/kg) (**Figure 2A**). Luzindole (LUZ) is a widely used non-selective antagonist of the melatonin receptors MT1 and MT2, enabling us to test whether the effects of exogenous melatonin were receptor-mediated (18, 31, 38, 39). As BALB/c mice are melatonin deficient but receptor competent (28), this model allows for the assessment of receptor-mediated versus receptor-independent effects of exogenous melatonin administration in the context of ILC2-driven allergic airways disease. In this model, melatonin and Luzindole were both dissolved in DMSO, and thus a single shared vehicle control (VEH) was used across all treatment groups.

Using the same multi-parameter approach applied to the AA model, we evaluated disease outcomes in IL-33 challenged mice in the presence or absence of melatonin and Luzindole. Lung-to-body weight ratios increased significantly only in IL-33 exposed mice, whereas mice receiving both melatonin and Luzindole showed no significant increase compared with PBS controls (**Figure 2B**). Similarly, total BALF cellularity was elevated in IL-33 challenged mice but notably not in those co-treated with melatonin and Luzindole (**Figure 2C**).

Histologically, melatonin did not fully prevent immune cell accumulation in the lung parenchyma when Luzindole was present, however, the extent of infiltration remained markedly reduced compared with IL-33 alone (**Figures 2D, E**). Epithelial thickening was likewise significantly lower in IL-33 challenged mice receiving either melatonin alone or melatonin and Luzindole (**Figures 2D, F**).

Flow cytometric analysis revealed that although intranasal IL-33 induced robust eosinophilia in both lung and BALF, melatonin significantly limited this increase (**Figures 2G, H**), even under melatonin receptor antagonism (**Figures 2G, H**). In this model, moderate increases in pulmonary neutrophilia are expected and were indeed observed in IL-33-treated mice (**Figures 2I, J**), however the degree of increase in neutrophil populations was markedly lower in mice receiving melatonin +/- Luzindole alongside IL-33 (**Figures 2I, J**). Notably, ILC2 proportions were comparable between IL-33 alone and IL-33 plus melatonin and Luzindole groups (**Figures 2K, L**), yet BALF concentrations of IL-5 (**Figures 2M**) and IL-13 (**Figures 2N**) remained significantly reduced with melatonin treatment, again indicating dampened ILC2 activity rather than altered ILC2 abundance.

Consistent with these findings, physiological measurements demonstrated that melatonin continued to improve airway function in the presence of Luzindole (**Figures 2O, P**), suggesting that receptor-independent mechanisms may contribute to melatonin’s protective effects in allergic airways disease.

### Melatonin regulates ILC2 function independently of canonical MT1/MT2 receptors

In both mice and humans, two canonical melatonin receptors, MT1 (*Mtnr1a*) and MT2 (*Mtnr1b*), have been described (18, 40–42), although neither has yet been reported to be expressed on ILC2s. Using publicly available single cell RNAseq datasets from both mouse and human studies we investigated the distribution of the genes for MT1 (*Mtnr1a*) and MT2 (*Mtnr1b*) across multiple organ systems. Our meta-analysis of 49 murine single-cell datasets revealed that *Mtnr1a* transcripts were detectable in the lung, whereas Mtnr1b was largely absent (**Supplementary Figure 3A**), whereas in a similar analysis of over 250 human datasets, both *Mtnr1a* and *Mtnr1b* were detected (**Supplementary Figure 3B**). Pulmonary ILC2s were identifiable in two human scRNAseq datasets; however, neither dataset showed detectable expression of *Mtnr1a* or *Mtnr1b* in these cells (**Supplementary Figure 3C**). The available murine datasets did not identify pulmonary ILC2s, however ILC2s were identified in the colon and small intestine (**Supplementary Figure 3D**), yet neither *Mtnr1a* nor *Mtnr1b* was detectable in these populations. We also looked for expression of genes involved in the synthesis of melatonin, namely *Aanat* and *Asmt*, but again found that although these are expressed in both the mouse and human lung, they were not found to be detected in ILC2 of either species (**Supplementary Figures 3A–D**). To further investigate the expression of *Mtnr1a* and *Mtnr1b* in pulmonary ILC2s we interrogated our bulk RNAseq dataset of purified, melatonin- and vehicle-treated murine ILC2s (**Supplementary Figure 3E**). Here, both *Mtnr1a* and *Mtnr1b* as well as *Aanat* and *Asmt* were again undetected, suggesting that ILC2s did indeed not express the genes for canonical melatonin receptors or those genes required for the synthesis of melatonin (**Supplementary Figure 3E**).

**Figure 3:**
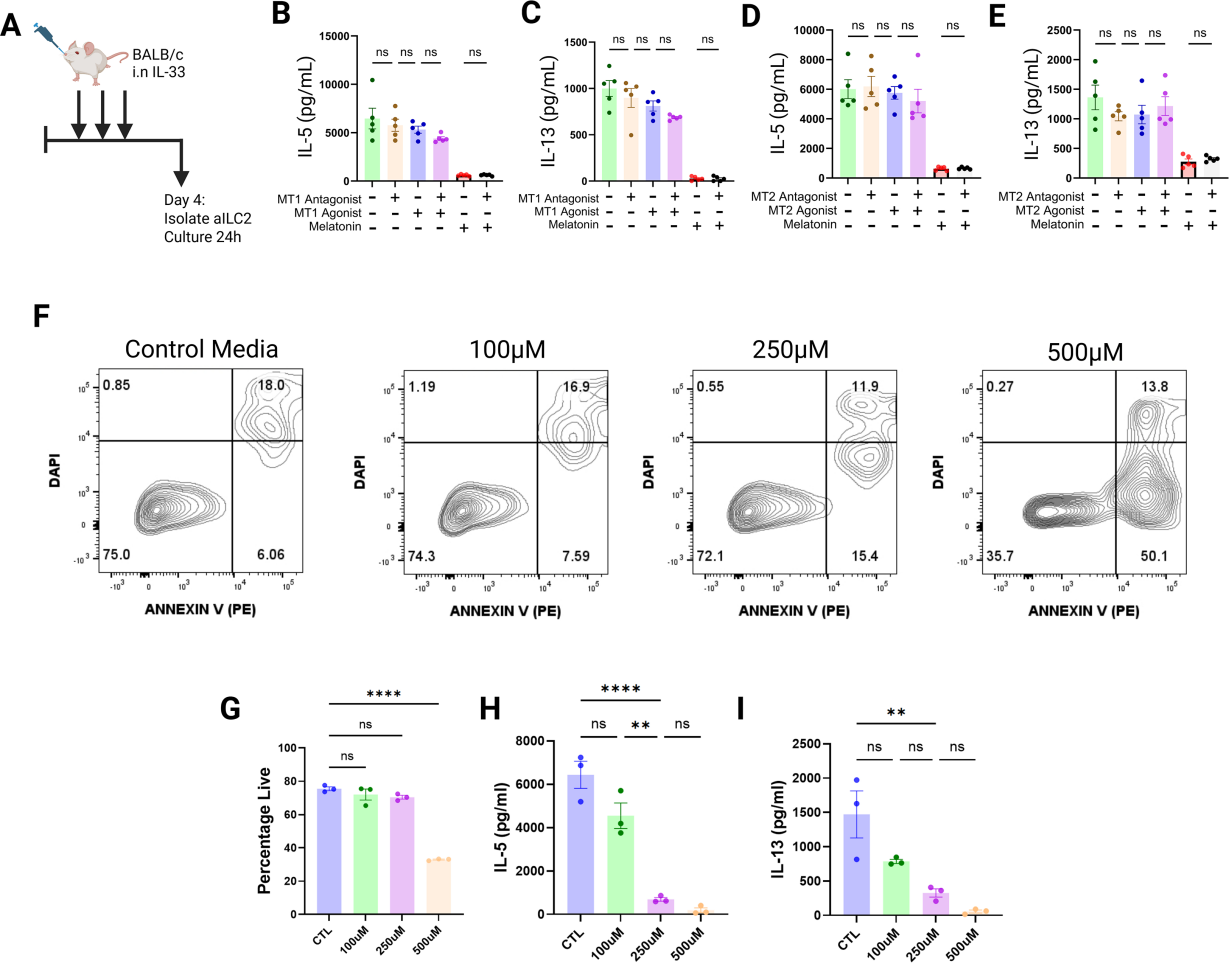
Melatonin regulates ILC2 function independently of MT1/MT2 receptors. **(A)** Schematic of the IL-33 challenge protocol. **(B, C)** IL-5 **(B)**, and IL-13 **(C)** concentrations in cell culture supernatant were quantified 24h after ILC2s were treated with either an MT1 agonist, MT1 antagonist, melatonin, or combination of these. **(D, E)** IL-5 **(D)**, and IL-13 **(E)** concentrations in cell culture supernatant were quantified 24h after ILC2s were treated with either an MT2 agonist, MT2 antagonist, melatonin, or combination of these. **(F)** Representative flow plots from apoptosis assay of melatonin treated ILC2s. **(G)** Quantification of live ILC2s deduced by Annexin V and DAPI staining as in **(F)** following *in vitro* treatment with varying concentrations of melatonin. **(H, I)** Quantification of IL-5 **(H)**, and IL-13 **(I)** in cell culture supernatant of ILC2s treated with various concentrations of melatonin. Data are mean ± SEM; n = 3–5 per group analyzed by one-way ANOVA with *post-hoc* Tukey’s t-tests. ns = P>0.05, **P< 0.01, ****P< 0.0001.

To corroborate these findings on a functional level, we isolated activated ILC2s from IL-33 challenged BALB/c mice (**Figure 3A**), which were then incubated for 24h with either an MT1 agonist (Ramelteon), MT1 antagonist (S-26131), MT2 agonist (Tasimelteon), MT2 antagonist (4-P-PDOT) and/or melatonin (**Figures 3B–E**). We found that pharmacological MT1 and MT2 agonism failed to replicate the effects of melatonin in suppressing IL-5 production (**Figures 3B, D**) or IL-13 production (**Figures 3C, E**). Additionally, we found that melatonin continued to have suppressive effects on ILC2 effector function even in the presence of MT1 and MT2 antagonists (**Figures 3B–E**).

Throughout our *in vitro* experiments, we exposed activated ILC2s to a pharmacological concentration (250µM) of melatonin as has been shown previously to be maximally effective in driving phenotypic changes in T-cells without causing toxicity (43). Indeed, for ILC2s we observed that this concentration did not precipitate changes in cellular viability (**Figures 3F, G**), and was additionally required to produce maximal suppression of ILC2 activity (**Figures 3H, I**). Whilst the concentration of melatonin used *in vitro* far exceeds that found *in vivo*, *in vitro* conditions lead to the rapid degradation of melatonin reducing its concentration swiftly over a 24h period. For this reason, a relatively high starting concentration was needed to reliably induce the phenotype being studied.

Together, these transcriptomic and functional data exclude a requirement for canonical MT1 or MT2 receptor signaling in melatonin-mediated regulation of ILC2 function.

### Melatonin exerts distinctly protective effects on ILC2-mediated AHR

**Figure 4:**
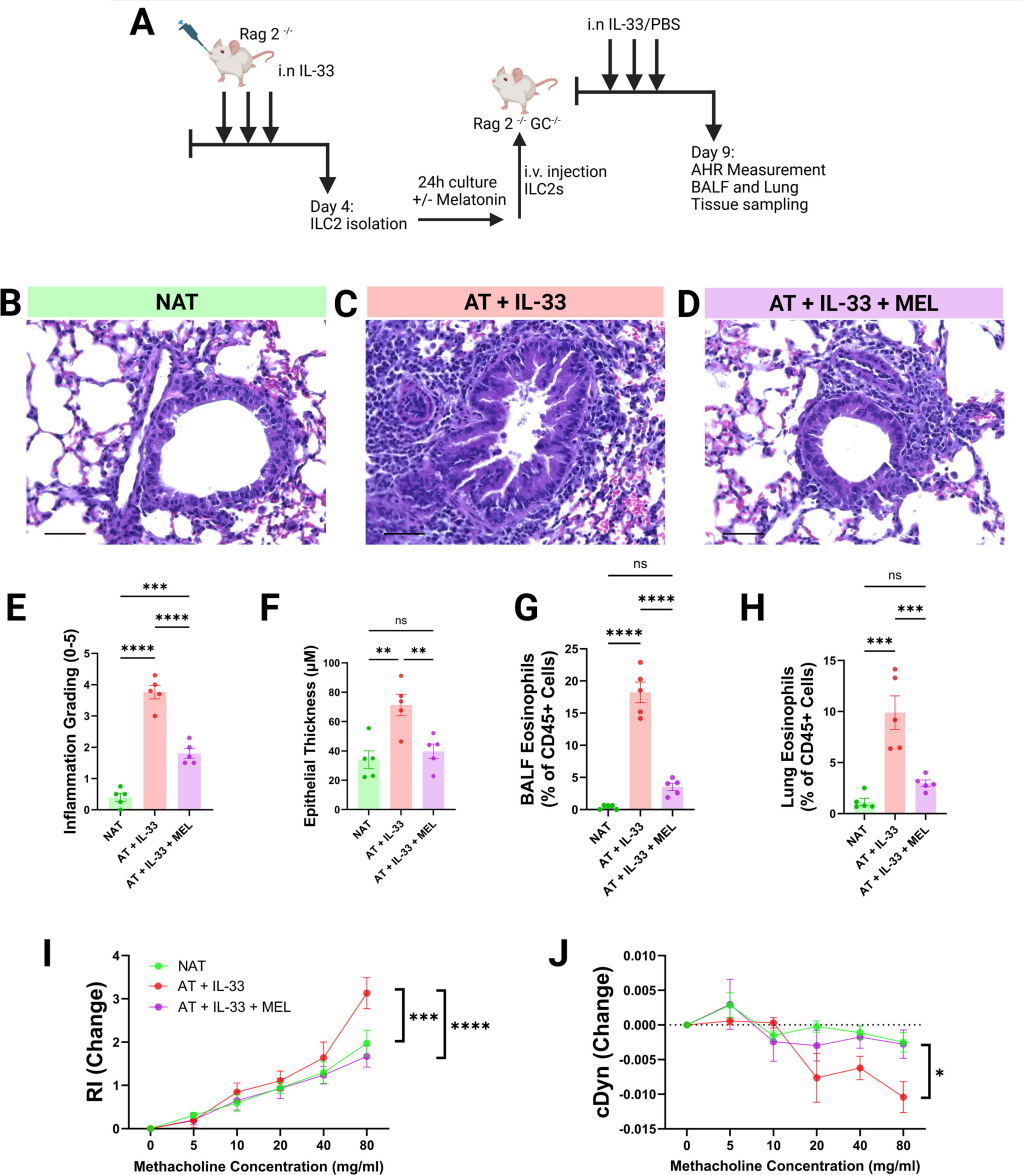
Melatonin exerts distinctly protective effects on ILC2-mediated airway hyperreactivity. **(A)** Schematic of the ILC2 adoptive transfer protocol. Rag2^-^/^-^ mice were intranasally challenged with IL-33 for three consecutive days, after which lungs were harvested and activated ILC2s (aILC2s) were isolated. aILC2s were cultured for 24 h in the presence or absence of melatonin (250 μM). On Day 5, 8 × 10^4^ aILC2s were adoptively transferred via tail vein injection into Rag2^-^/^-^Il2rg^-^/^-^ mice, which were subsequently challenged with IL-33 for three days in the presence or absence of melatonin. On Day 9, AHR measurements were recorded and tissues were collected. **(B–D)** Representative H&E-stained lung sections from no-transfer controls **(B)**, adoptive transfer + IL-33 mice **(C)**, and adoptive transfer + IL-33 + melatonin mice **(D)** (40× magnification; scale bar = 100 μm). **(E)** Inflammation scores from H&E-stained FFPE lung sections (mean of three independent blinded scorers). **(F)** Airway epithelial thickness (eight measurements per airway, ten airways per mouse, analyzed in QuPath). **(G, H)** BALF eosinophils **(G)** and lung eosinophils **(H)** quantified by flow cytometry as a proportion of total CD45^+^ cells. **(I, J)** Airway resistance **(I)** and dynamic compliance **(J)** in response to methacholine. Data are mean ± SEM; n = 5 per group. One-way ANOVA was used for single-endpoint comparisons; methacholine dose–response curves were analyzed by two-way ANOVA with Tukey’s *post hoc* multiple comparisons test.

Building on our observations that melatonin offered protective effects, restraining the emergence of airway hyperreactivity in our murine models of asthma, we next aimed to identify if this could be applied more specifically to ILC2 activity *in vivo*. ILC2s are capable of both initiating and propagating many of the pathological features of allergic airways disease, potentially playing a critical role in acute disease exacerbations (44–47). To interrogate whether melatonin could restrain ILC2 effector function we employed an adoptive transfer model, whereby activated ILC2s were isolated from Rag2^-/-^ mice, cultured for 24h in the presence or absence of melatonin, and then adoptively transferred the ILC2s into Rag2^-/-^GC^-/-^ mice utilizing established protocol as described by our group previously (48, 49) (**Figure 4A**). Following the adoptive transfer, each mouse was intranasally challenged with 0.5 µg rmIL-33 for 3 days prior to AHR measurements and tissue sampling being performed (**Figure 4A**). Using this model, we found that whilst adoptively transferred activated ILC2s were indeed capable of inducing pathology in mice lacking an adaptive immune system, melatonin treatment significantly restrained this.

Histological examination of lung tissue from IL-33 challenged Rag2^^-^/^-^^GC^^-^/^-^^ mice revealed that adoptive transfer of activated ILC2s induced marked immune cell infiltration into the lung parenchyma and significant thickening of the airway epithelium compared with no-transfer controls (**Figures 4B, C, E, F**). In contrast, these pathological changes were largely abrogated in mice receiving melatonin-treated ILC2s (**Figures 4D–F**). Consistently, melatonin treatment of ILC2s significantly restrained the development of lung and BALF eosinophilia in this model (**Figures 4G–H**). Physiological assessment of airway function demonstrated that only vehicle-treated ILC2s induced airway hyperreactivity, whereas melatonin-treated ILC2s prevented the development of AHR following IL-33 challenge (**Figures 4I–J**).

Together, these data demonstrate a unique role for ILC2s in both initiating allergic airways disease and in responding to cues such as melatonin to dampen their effector phenotypes.

### Melatonin reprograms the effector state of activated ILC2s

**Figure 5:**
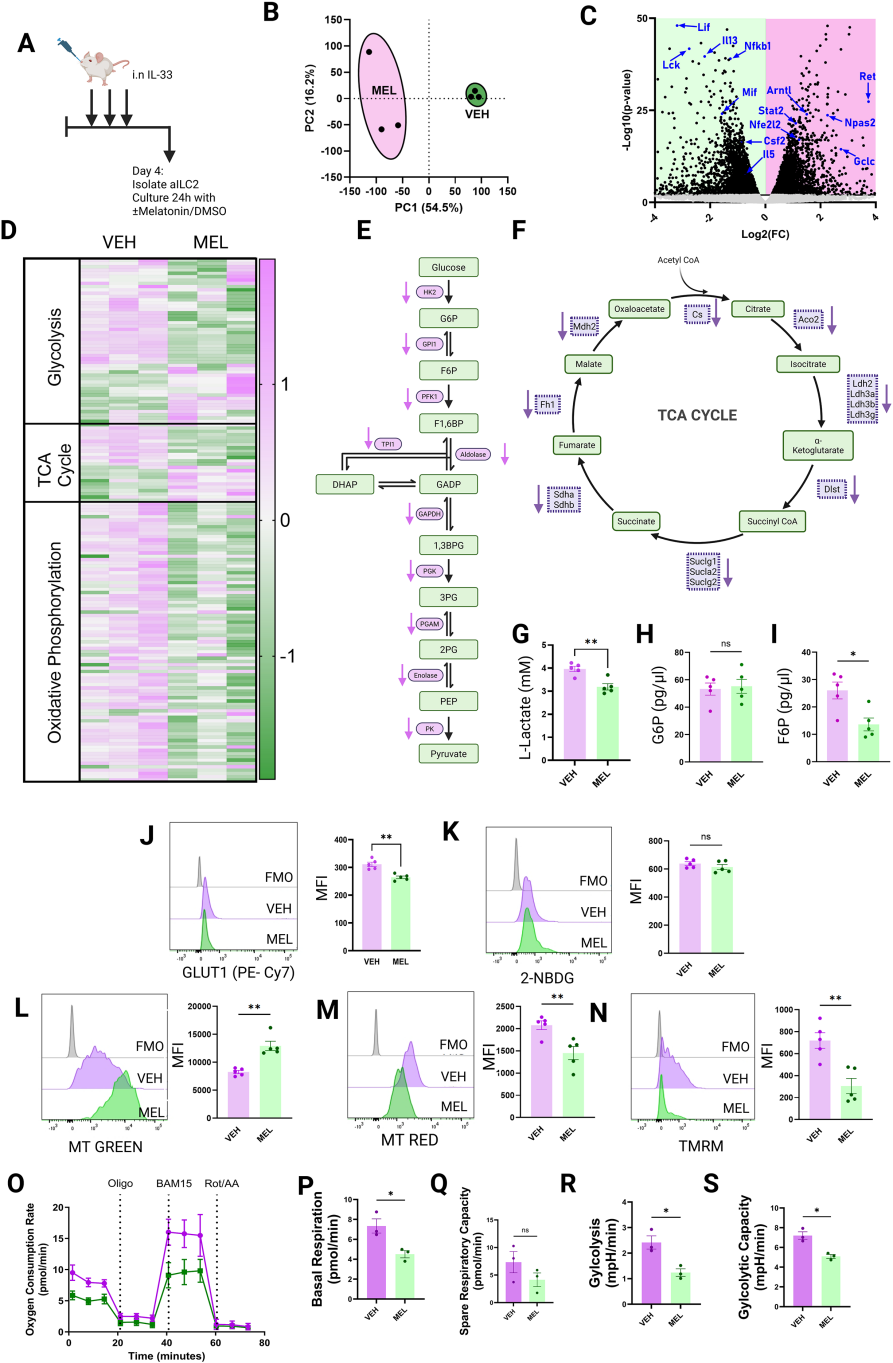
Melatonin reprograms ILC2 activity and metabolism. **(A)** Activated ILC2s (aILC2s) were collected from IL-33–challenged BALB/c mice and cultured for 24 h with melatonin or DMSO (vehicle control) prior to RNA extraction for bulk transcriptomic analysis. **(B)** PCA plot showing two main sources of variation between all samples. **(C)** Volcano plot comparing melatonin-treated and vehicle-treated aILC2s. **(D)** Heatmap of genes involved in cellular respiration (glycolysis, TCA cycle, oxidative phosphorylation) in melatonin-treated versus vehicle-treated aILC2s. **(E, F)** Diagrammatic overviews of glycolysis **(E)** and the TCA cycle **(F)**, with arrows indicating the direction of gene expression changes in melatonin-treated versus vehicle-treated ILC2s. **(G)** Enzymatic quantification of lactate accumulation in culture supernatants. **(H, I)** Glucose-6-phosphate **(H)** and fructose-6-phosphate **(I)** levels in cell extracts from cultured ILC2s. **(J)** Representative flow cytometry plots of GLUT1 expression on ILC2s ± melatonin, with quantification of median fluorescence intensity (MFI). **(K)** Glucose uptake measured by 2-NBDG staining and quantified as MFI. **(L)** Mitochondrial size assessed by MitoTracker Green staining and quantified as MFI. **(M, N)** MitoTracker Red **(M)** and TMRM **(N)** staining used to assess mitochondrial membrane polarization and quantified as MFI. **(O–S)** Mitochondrial respiratory profile showing oxygen consumption rates in response to sequential injections of oligomycin, BAM15, and rotenone + antimycin **(A)** Basal respiration **(P)**, spare respiratory capacity **(Q)**, and OCR-derived surrogate measures of glycolysis **(R)** and glycolytic capacity **(S)** are shown. Data are mean ± SEM; n = 5 per group **(G–N)** and n = 3 per group **(P–S)**. Two-tailed Student’s t-tests were used for pairwise comparisons. ns = P>0.05, *P< 0.05, **P< 0.01.

To further dissect the effects we had observed, we isolated aILC2s from IL-33 challenged BALB/c mice (**Supplementary Figures 2**) and performed bulk RNA sequencing, comparing the transcriptome of vehicle and melatonin treated cells (**Figure 5A**). Principal component analysis revealed that melatonin treatment induced distinct variations in the transcriptomic profile of ILC2s (**Figure 5B**). We found that melatonin treatment was associated with a significant increase in the expression of 3957 genes and a decrease in the expression of 4056 others (**Figure 5C**). Notably, pro-inflammatory genes such as *Il13, Il5, Csf2* among others were consistently downregulated following melatonin treatment (**Figure 5C**). Notable upregulated genes in melatonin-treated ILC2s were components of the molecular circadian clock such as *Arntl*, along with genes involved in glutathione synthesis such as *Gclc* (**Figure 5C**).

To further interrogate the anti-inflammatory action of melatonin treatment on ILC2s we cultured ILC2s ex vivo for 24h with either melatonin or vehicle control. Whilst we found that ex vivo vehicle treated cells produced high levels of cytokines, including IL-5, IL-13, IL-9 (**Supplementary Figures 4B–D**), and modest amounts of IL-10 and IL-6 (**Supplementary Figures 4E, F**), melatonin treatment greatly reduced the concentration of all cytokines analyzed (**Supplementary Figures 4B – F**). We next examined KLRG1 expression, a marker of mature, highly cytokine-producing ILC2s (**Supplementary Figures 4G**) (50, 51). Melatonin treatment significantly reduced KLRG1 expression, further indicating a shift away from a pathogenic effector state (**Supplementary Figures 4G**). Moreover, intracellular staining for IL-5 and IL-13 (**Supplementary Figures 4H–I**), corroborated with our previous findings that melatonin ILC2s produced less cytokine than vehicle treated controls (**Supplementary Figures 4B–F, H, I**). Expression of GATA-3, the master regulator of ILC2 identity and function (50, 52), was found to not be markedly affected by melatonin treatment (**Supplementary Figures 4J**). This indicates that melatonin alters ILC2 effector state without disrupting lineage identity.

### Melatonin reprograms metabolism and impairs mitochondrial function in activated ILC2s

Immune cells often rely on the coalescence of diverse metabolic pathways, which provide cells with the energy, reactive metabolites and essential cofactors needed to sustain high levels of activity in pro-inflammatory states. Given the central role of cellular metabolism in sustaining ILC2 effector function, we next examined how melatonin alters ILC2 bioenergetics. In our bulk RNAseq data, we noted that pathways such as oxidative phosphorylation and electron transport chain were significantly downregulated (**Supplementary Figures 5B, C**). We interrogated gene expression changes in more detail focusing on pathways involved in cellular glucose handling: Glycolysis, TCA Cycle, Oxidative Phosphorylation (**Figures 5D–F**). We found that melatonin treatment precipitated major transcriptomic shifts in ILC2s whereby genes encoding the key enzymes in glycolysis (**Figure 5E**) and the TCA cycle (**Figure 5F**) were expressed at demonstrably lower levels compared to controls.

Consistent with transcriptomic suppression of glycolytic and mitochondrial pathways, melatonin-treated ILC2s exhibited marked functional alterations in glucose utilization and oxidative metabolism (**Figures 5G–S**). Firstly, to quantify the glycolytic activity of ILC2s ex-vivo, we analyzed their production of L-lactate, a terminal metabolite produced in the glycolysis pathway, noting that melatonin treatment precipitated a marked decrease in concentration (**Figure 5G**). We also quantified metabolites involved in the most proximal stages of the glycolysis pathway, namely glucose-6-phosphate (G6P) and fructose-6-phosphate (F6P) (**Figures 5H, I)**. Whilst we found that melatonin did not affect the concentration of G6P (**Figure 5H**), it did seem to greatly reduce the amount of F6P present in our cell samples, indicating potentially that the key disruption to this pathway occurred at the step where G6P is converted to F6P (**Figure 5E**).

Our results indicated that melatonin suppresses glycolysis in ILC2s, but it remained unclear whether this effect was due to impaired glucose uptake. To address this, we assessed surface expression of the glucose transporter GLUT1 on ILC2s by flow cytometry (**Figure 5J**). Although melatonin treatment was associated with reduced GLUT1 expression (**Figure 5J**), this did not translate into a functional deficit in glucose uptake, as melatonin-treated cells displayed comparable 2-NBDG uptake to controls (**Figure 5K**).

In parallel, we analyzed transcriptomic changes in the β-oxidation pathway, a major route for fatty acid utilization in ILC2s (**Supplementary Figures 6A, B**). Melatonin treatment was associated with broad suppression of multiple genes involved in fatty acid breakdown (**Supplementary Figure 6B**). To determine whether these transcriptomic changes were reflected at the metabolic level, we quantified β-hydroxybutyrate, a terminal product of β-oxidation, in both cell lysates and culture supernatants. In both compartments, melatonin treatment prevented the accumulation of β-hydroxybutyrate observed in vehicle-treated cells (**Supplementary Figures 6C, D**). Because β-oxidation directly feeds into the TCA cycle, this suppression is likely to further contribute to the marked reduction in TCA cycle activity seen in melatonin-treated ILC2s (**Figure 5F**).

Whilst minor amounts of energy can be produced in the cytoplasm during glycolysis, the major source of cellular energy comes from oxidative phosphorylation, which occurs in the mitochondria. To assess the impact of melatonin treatment on the mitochondria of ILC2s we performed a range of assays to quantify mitochondrial size and membrane potential (**Figures 5L–M**). Our MitoTracker Green assay (MT Green) revealed that following treatment with melatonin, ILC2 mitochondria increased in size (**Figure 5L**) whilst MitoTracker Red (MT Red) and TMRM assays suggested that mitochondrial membranes were significantly depolarized following melatonin treatment (**Figures 5M, N**). Taken together, these assays suggested that melatonin induces mitochondrial reprogramming in ILC2s.

Given these transcriptomic and biochemical changes, we next asked whether melatonin altered real-time cellular metabolism. Metabolic profiling by Seahorse assay suggested that melatonin treated ILC2s exhibited decreased rates of basal respiration (**Figure 5P**), decreased spare respiratory capacity (**Figure 5Q**), and both reduced active rates of glycolysis (**Figure 5R**) as well as reduced glycolytic capacity (**Figure 5S**).

Overall, our data demonstrate that melatonin profoundly disrupts ILC2 metabolism. Although glucose uptake remains intact, downstream utilization of both glucose and fatty acids is markedly impaired, coinciding with reduced mitochondrial membrane potential, diminished respiratory capacity, and blunted glycolytic flux. Together, these findings indicate that melatonin drives ILC2s into a metabolic state incompatible with a pro-inflammatory effector phenotype.

### Melatonin drives glutathione accumulation in ILC2s

Whilst we noted that melatonin suppressed glycolysis in ILC2s (**Figure 5E**), we also found that glucose uptake remained unchanged (**Figure 5K**). Moreover, intracellular levels of glucose-6-phosphate (G6P) were not reduced following melatonin treatment (**Figure 5H**), whereas fructose-6-phosphate (F6P) was markedly decreased (**Figure 5I**). Together, these findings suggest a diversion of glucose flux away from glycolysis at this critical branch point in the pathway. Importantly, this junction also represents the entry point into the pentose phosphate pathway (PPP), which shunts glucose away from energy production and instead generates NADPH, an essential cofactor required for glutathione synthesis and redox homeostasis.

**Figure 6:**
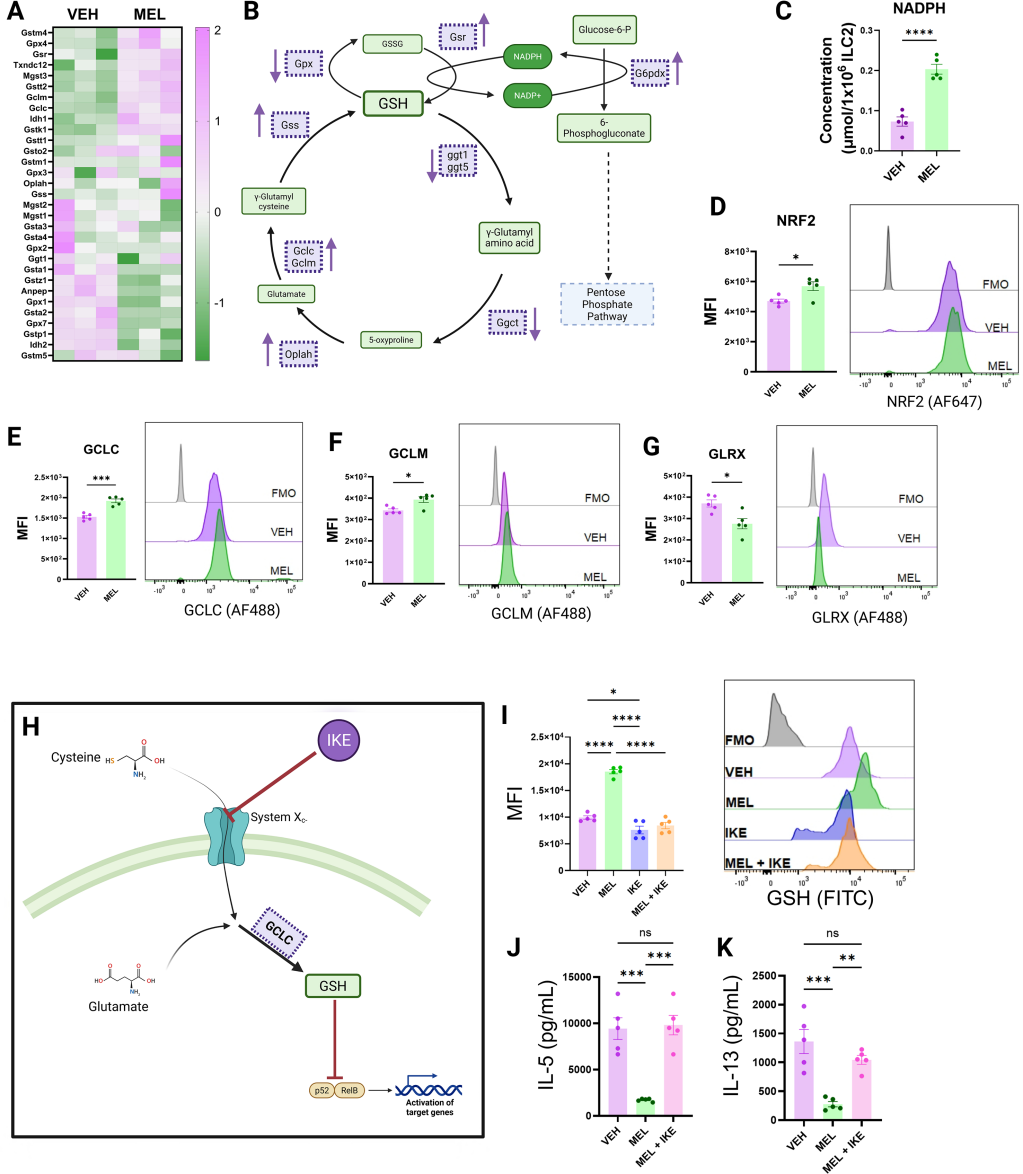
Melatonin drives glutathione accumulation in ILC2s. **(A)** Heatmap of genes associated with glutathione synthesis. **(B)** Diagrammatic representation of the glutathione synthesis pathway, with arrows indicating the direction of change in key genes in melatonin-treated versus vehicle-treated ILC2s. **(C)** NADPH concentrations quantified in cell lysates of melatonin-treated and vehicle-treated ILC2s. **(D–G)** NRF2 **(D)**, GCLC **(E)**, GCLM **(F)**, and GLRX **(G)** expression levels quantified by flow cytometry and expressed as median fluorescence intensity (MFI). **(H)** Schematic overview of the mechanism of action of IKE, which blocks the cystine/glutamate antiporter System Xc^-^ to limit glutathione synthesis. **(I)** Glutathione levels in live ILC2s treated with vehicle, melatonin, melatonin + IKE, or IKE alone, quantified by fluorescent staining and expressed as MFI. **(J, K)** IL-5 **(J)** and IL-13 **(K)** concentrations in culture supernatants of ILC2s treated with vehicle, melatonin, or melatonin + IKE. **(A–K)** Data are mean ± SEM; n = 5 per group. Student’s two-tailed t-tests or one-way ANOVA with Tukey’s *post hoc* multiple comparisons tests were used, as appropriate.

Consistent with this, our RNAseq analysis revealed significant alterations in the expression of genes involved in glutathione metabolism following melatonin treatment (**Figure 6A**). Notably, genes promoting glutathione biosynthesis were broadly upregulated, whereas genes associated with glutathione consumption or turnover were comparatively reduced (**Figure 6B**). Furthermore, expression of G6pd was significantly increased in melatonin-treated ILC2s (**Figure 6B**). G6PD catalyzes the rate-limiting step of the PPP, effectively diverting G6P away from glycolysis toward 6-phosphogluconate, thereby promoting NADPH generation and glutathione synthesis (**Figure 6B**). To determine whether this metabolic shift translated into increased cellular reducing capacity, we measured intracellular NADPH levels in ILC2s following melatonin treatment (**Figure 6C**). Melatonin-treated cells exhibited a significant increase in NADPH concentration relative to controls (**Figure 6C**), providing a biochemical basis for enhanced glutathione synthesis.

From our RNAseq analysis, we noted that many of the upregulated genes in the glutathione biosynthesis pathway (including *Gclc* and *Gclm*) are canonical targets of the transcription factor NRF2 (24, 53, 54). Subsequent flow cytometric analysis of NRF2 expression in ILC2s revealed that melatonin treatment significantly increased NRF2 protein levels (**Figure 6D**). To further validate our transcriptomic findings at the protein level, we assessed expression of key enzymes involved in glutathione biosynthesis, including GCLC, GCLM, and GLRX (**Figures 6E - G**). Consistent with enhanced glutathione production, melatonin-treated ILC2s exhibited increased expression of GCLC and GCLM, enzymes that catalyze the rate-limiting steps in glutathione synthesis (**Figures 6E, F**). In contrast, expression of GLRX, which promotes the conversion of active reduced glutathione (GSH) into its oxidized form (GSSG), was significantly decreased following melatonin treatment (**Figure 6G**).

We hypothesized that because glutathione synthesis depends on cysteine availability (24), restricting cysteine import would abrogate the metabolic and functional effects of melatonin on ILC2s (**Figure 6H**). To test this, we inhibited the cysteine/glutamate antiporter System Xc^-^ using Imidazole Ketone Erastin (IKE), thereby limiting substrate availability for GSH synthesis (**Figure 6H**). Using a flow cytometry-based assay for intracellular glutathione, we found that although melatonin increased cellular GSH levels, this effect was lost when cells were co-treated with melatonin and IKE (**Figure 6I**).

Strikingly, this accumulation of glutathione proved functionally critical for the suppression of ILC2 activity. Whereas melatonin alone significantly reduced ex vivo production of IL-5 (**Figure 6J**) and IL-13 (**Figure 6K**). Importantly, blockade of cysteine-dependent glutathione synthesis was sufficient to reverse melatonin-mediated suppression of ILC2 cytokine production (**Figures 6J, K**). Together, these data indicate that melatonin suppresses ILC2 effector function in a glutathione-dependent manner.

### Melatonin suppresses ILC2 function via NRF2 activation

**Figure 7:**
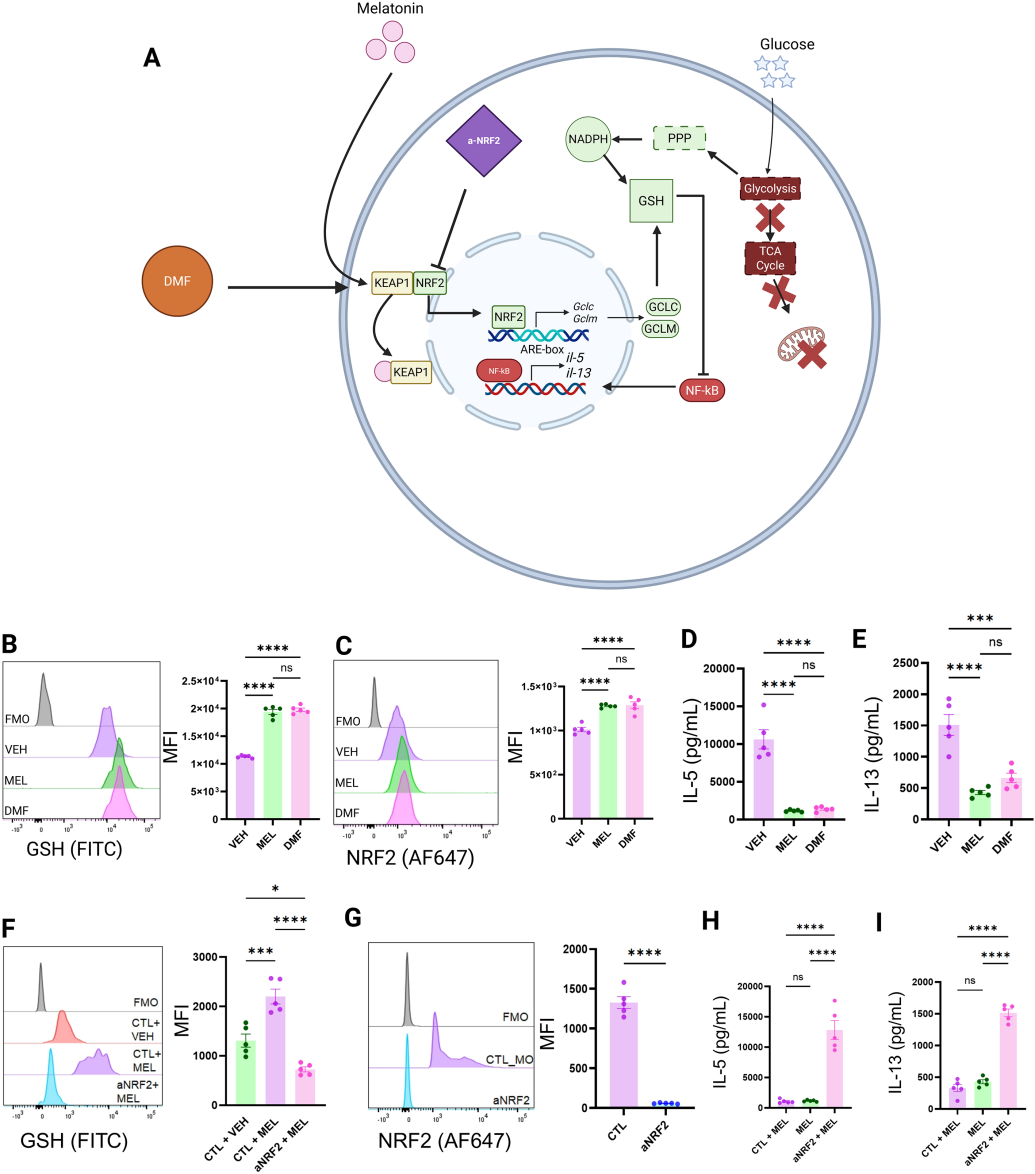
Melatonin suppresses ILC2 function via NRF2 activation. **(A)** Schematic overview of the Melatonin-NRF2-Glutathione axis and its relationship with cellular glucose handling. Dimethyl fumarate (DMF), an FDA-approved therapeutic, drives NRF2 activity by releasing it from KEAP1. An a-NRF2 morpholino was used to selectively disrupt the translation *Nfe2l2*, functionally eliminating the action of NRF2. **(B)** Intracellular glutathione (GSH) levels were assessed by flow cytometry in melatonin and DMF treated ILC2s. **(C)** The expression of NRF2 was assessed by flow cytometry in melatonin and DMF treated ILC2s. IL-5 **(D)** and IL-13 **(E)** concentrations in ILC2 cell culture supernatants were quantified following treatment with melatonin and DMF. **(F)** Intracellular GSH levels were assessed by flow cytometry following treatment with control morpholino, DMSO vehicle, melatonin, and a-NRF2 morpholino. **(G)** Quantification of NRF2 expression levels in ILC2s treated with either control morpholino or aNRF2 morpholino. IL-5 **(H)** and IL-13 **(I)** concentrations were quantified from ILC2 cell culture supernatants following treatment with melatonin and either a control morpholino or aNRF2 morpholino. **(B–I)** Data are mean ± SEM; n = 5 per group analyzed by two-tailed Student’s t-tests or one-way ANOVA with Tukey’s *post hoc* multiple comparisons tests as appropriate. ns = P>0.05, *P< 0.05, ***P< 0.001, ****P< 0.0001.

Whilst we found that melatonin’s immunosuppressive effects on ILC2s were glutathione dependent, the upstream mechanism linking melatonin to glutathione biosynthesis remained unclear. Dimethyl fumarate (DMF), an FDA-approved drug prescribed for the treatment of multiple sclerosis (55, 56), activates NRF2 by disrupting its interaction with KEAP1 (**Figure 7A**), allowing NRF2 to translocate to the nucleus and become transcriptionally active (55–57). DMF, via the covalent modification of KEAP1 cysteine residues, leads to reliable NRF2 stabilization, with dose-dependent effects that are cytoprotective at low doses. Unlike many experimental NRF2 activators (e.g., synthetic electrophiles or natural compounds like sulforaphane), DMF has undergone extensive Phase III trials, demonstrating its efficacy and safety in humans. We therefore used DMF as a pharmacological tool to activate NRF2 independently of melatonin. We hypothesized that if melatonin suppresses ILC2s via NRF2 activation, then pharmacological activation of NRF2 with DMF should phenocopy the effects of melatonin.

Indeed, our intracellular GSH detection assay revealed that both treatment with DMF and melatonin induced increases in ILC2 GSH levels (**Figure 7B**). Similarly, treatment with both DMF and melatonin was associated with greater levels of intracellular NRF2 expression detectable by flow cytometry (**Figure 7C**). Concurrent with these findings we also noted that DMF treatment precipitated a similar suppression in ILC2 activity with significant reductions in IL-5 and IL-13 concentrations being detected in the supernatant of ILC2s (**Figures 7D, E**).

Next, we isolated activated ILC2s from the lungs of IL-33 challenged mice, subsequently treating them with morpholinos to block the translation of NRF2 whilst treating them with melatonin (**Figures 7A, G**). We found that blocking the translation of NRF2 prevented GSH accumulation (**Figure 7F**) as well as negated the immunosuppressive action of melatonin treatment (**Figures 7H, I**). These results suggest that NRF2 is a necessary component of melatonin signaling pathways in ILC2s, linking the canonical circadian hormone to cellular redox machinery.

Together, these findings position NRF2 as the central mediator of melatonin-induced metabolic reprogramming and functional suppression in ILC2s and identify NRF2 as a therapeutically actionable target.

### Melatonin exerts immunomodulatory effects on human ILC2s in a glutathione dependent manner

Encouraged by our murine data, which demonstrated that melatonin reprograms ILC2 metabolism and enhances glutathione-dependent immunosuppression, we sought to determine whether these mechanisms also operate in human ILC2s.

**Figure 8:**
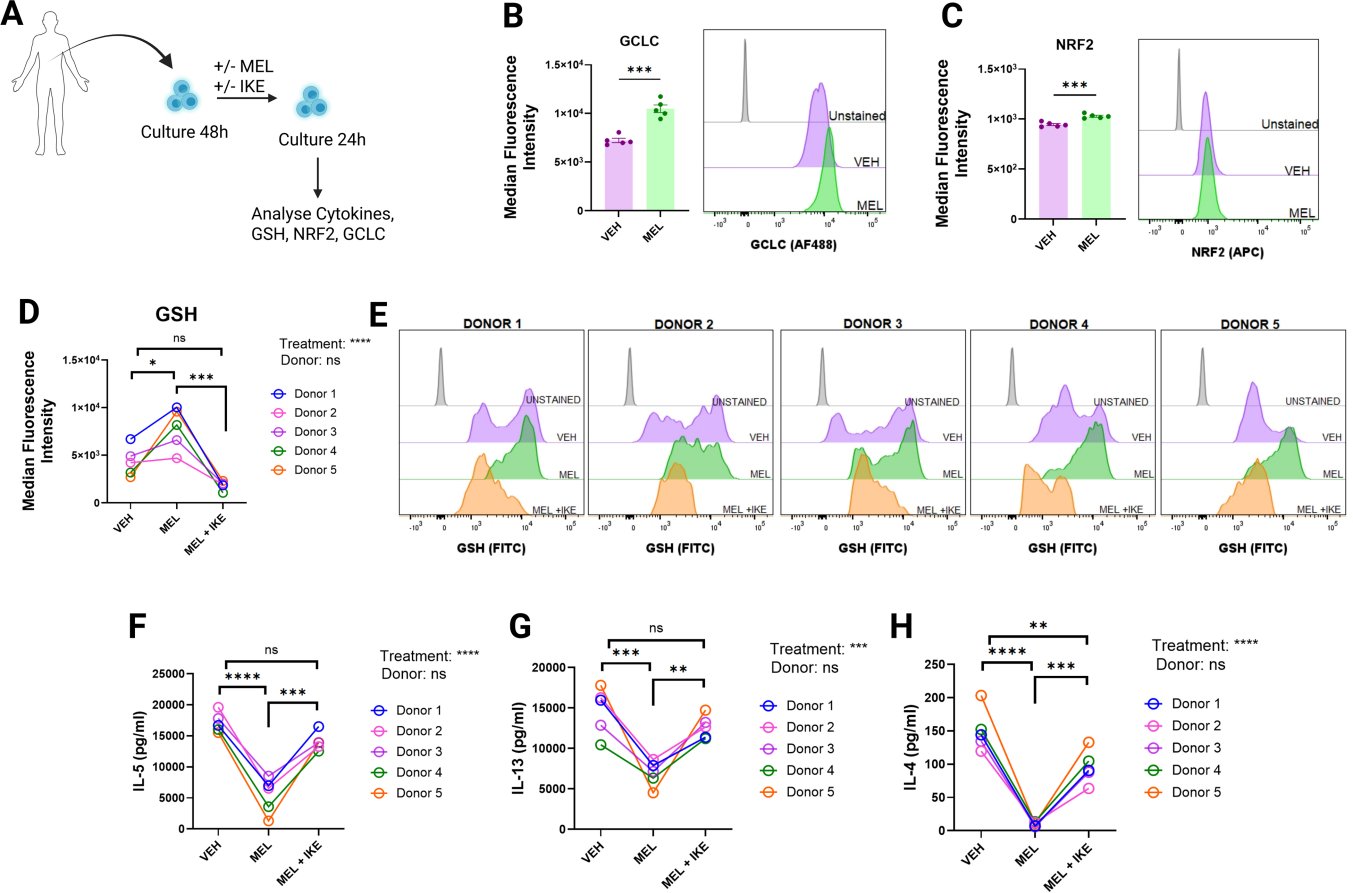
Melatonin exerts immunomodulatory effects on human ILC2s in a glutathione dependent manner. **(A)** Schematic overview of the experimental design. Human ILC2s were isolated and cultured for 48 h, followed by treatment with melatonin and IKE for 24 h. **(B, C)** Flow cytometric quantification of intracellular GCLC **(B)** and NRF2 **(C)** expression in human ILC2s following melatonin treatment. **(D)** Quantification of intracellular glutathione levels in human ILC2s following treatment with melatonin and IKE. **(E)** Representative flow cytometry plots from each donor showing glutathione detection in human ILC2s following melatonin and IKE treatment. **(F–H)** IL-5 **(F)**, IL-13 **(G)**, and IL-4 **(H)** concentrations in culture supernatants from melatonin-treated and melatonin + IKE–treated human ILC2s. **(B–H)** Data are mean ± SEM; n = 5 independent donors. Data were analyzed by two-tailed Student’s t-tests or two-way ANOVA with Tukey’s *post hoc* multiple comparisons tests, as appropriate. Two-way ANOVA factors were Treatment and Donor.

To explore this, we isolated human ILC2s, activated them *in vitro*, and treated them with melatonin, with or without the cysteine import inhibitor IKE (**Figure 8A**). Consistent with murine ILC2s, melatonin increased human ILC2 expression of GCLC, the rate-limiting enzyme in glutathione synthesis, as well as NRF2 (**Figures 8B, C**). Intracellular GSH levels were elevated in all five donor samples following melatonin treatment (**Figures 8D, E**), whereas co-treatment with IKE prevented this accumulation (**Figures 8D, E**). Functionally, glutathione was essential for melatonin-mediated immunosuppression, as IKE co-treatment abolished the reductions in IL-5, IL-13, and IL-4 production observed with melatonin alone (**Figures 8F–H**). These data indicate that melatonin-dependent NRF2 activation and glutathione-mediated suppression of effector function are conserved between murine and human ILC2s.

## Discussion

Circadian variation in asthma severity has been recognized for decades, yet the hormonal mechanisms underlying this phenomenon remain incompletely understood. In this study, we sought to elucidate the role of melatonin in regulating ILC2 metabolism and effector function, in the context of allergic airways disease. We propose that melatonin acts as a metabolic modulator in ILC2s, redirecting glucose utilization toward redox-supporting pathways that restrain inflammatory effector programs.

In our model of allergen-induced allergic airways disease we noted a strikingly beneficial effect associated with melatonin treatment whereby pulmonary eosinophilia and airway hyperreactivity were significantly reduced. In this model we exposed BALB/c mice to *Alternaria alternata* and treated them with melatonin both at 17:00, since this is a time point that has been found to result in the most severe induction of features of allergic airway pathology (9, 27). In this study the mice used, have been reported to be deficient in endogenous melatonin production, as are C57BL/6 mice, but importantly are still able to express canonical melatonin receptors (28, 42). Comparing the effects seen here to a model using a melatonin-competent mouse, for example from the CBA strain, would be beneficial for future studies that consider in more detail the phasing of melatonin treatment *in vivo*. Physiological concentrations of melatonin are orders of magnitude lower than the pharmacological doses used in this study. For example, in melatonin competent mice (e.g. CBA mice) the peak in pineal melatonin is usually in the pM range. In humans, this is similar, with peaks generally being around 150pg/L in the serum. The exact minimum effective concentration that recapitulates our observed suppressive ILC2 phenotype *in vivo*, remains to be determined, and will likely rely on a complex grouping of factors that take into account systems with endogenous melatonin rhythms (such that were absent in our studies both *in vitro* and *in vivo* with BALBc mice). Nevertheless, our findings demonstrate that exogenous melatonin can modulate ILC2 function even in a melatonin-deficient background.

Whilst studies in humans have demonstrated that patients with asthma exhibit dysregulated rhythms in serum melatonin concentrations, the pathological mechanisms precipitated by this remain poorly understood (15). Part of the previously identified dysregulation is a phase shift in which peak melatonin concentrations occur significantly later in patients with asthma than in healthy individuals (15). Given our finding that NRF2 is a critical target for melatonin, we considered whether the gene *Nfe2l2* was expressed in a rhythmic fashion. Indeed, previous studies indicate that *Nfe2l2*, along with NRF2 target genes such as *Gclc* and *Gclm*, exhibits circadian oscillations in the lung, as well as kidney, and aorta (58, 59). Consistent with this, pulmonary glutathione levels also display marked circadian variation, suggesting tight circadian control of this antioxidant pathway (59). Together, these observations support a model in which pathological consequences of altered melatonin rhythms in asthma may arise from a temporal mismatch between melatonin availability and NRF2-driven redox programs in key immune cell populations such as ILC2s. In this context, disruption of temporal coordination, rather than hormone abundance alone, may limit effective engagement of melatonin-dependent immunoregulatory pathways.

Previous studies indicate that airway epithelial cells can respond to melatonin via MT1 to reduce alarmin production (18), whereas airway smooth muscle hyperreactivity may be enhanced through MT2 signaling (60). To distinguish potential receptor-dependent and receptor-independent effects of melatonin, we employed Luzindole, a dual MT1/MT2 antagonist. Given that melatonin is a small amphiphilic molecule (61), its intracellular actions do not necessarily require engagement of surface receptors. To directly assess whether MT1 or MT2 mediate melatonin’s effects in ILC2s, we treated cells with selective MT1 and MT2 agonists and antagonists. Neither class of agonist recapitulated the effects of melatonin, nor did either antagonist abrogate melatonin-induced changes in ILC2 phenotype or function. Together, these findings suggest that MT1 and MT2 are not functionally relevant in ILC2s. Consistent with this, a meta-analysis of publicly available RNA-seq datasets revealed no detectable expression of either *Mtnr1a* or *Mtnr1b* in ILC2 populations. Definitive genetic validation using MT1- or MT2-deficient ILC2s remains an important next step in confirming that melatonin acts on ILC2s independent of canonical melatonin receptors.

Several studies have proposed a direct biochemical interaction between melatonin and the NRF2 pathway. Under homeostatic conditions, NRF2 is sequestered in the cytoplasm through its interaction with the repressor protein KEAP1. Melatonin has been reported to bind KEAP1, thereby disrupting this interaction and permitting NRF2 to translocate to the nucleus where it can activate antioxidant response element (ARE)-driven gene expression (25, 41, 62, 63). Consistent with this model, NRF2 activation promotes transcription of genes involved in redox homeostasis and glutathione biosynthesis, including *Gclc* and *Gclm* (41, 63). In our study, melatonin-treated ILC2s exhibited increased intracellular NRF2 staining, consistent with enhanced availability of active NRF2. In parallel, we observed upregulation of canonical NRF2 target genes such as *Gclc* and *Gclm*, supporting the conclusion that melatonin augments NRF2 transcriptional activity in ILC2s. Whilst previous work by Nagashima et al. has elegantly demonstrated that NRF2 is a key suppressor of ILC2 activation, our study provides the missing upstream link, identifying melatonin, a canonical circadian hormone, as a critical activator of the NRF2-Glutathione axis in ILC2s (64).

Initial analysis of our RNA-seq dataset comparing melatonin- and vehicle-treated ILC2s revealed broad suppression of pro-inflammatory gene expression following melatonin treatment, including canonical type 2 cytokines such as *Il5*, *Il13*, and *Csf2*. These findings align with previous reports that melatonin reduces ILC2-derived IL-5 and IL-13 in oxazolone-induced colitis models (65), suggesting a conserved anti-inflammatory effect of melatonin across tissues. Notably, we also observed a marked reduction in KLRG1 expression following melatonin treatment. Because KLRG1 is widely used as a marker of highly activated, cytokine-producing ILC2s (66, 67), this finding supports the idea that melatonin drives a phenotypic transition from an activated, KLRG1^high^ effector state toward a more quiescent, KLRG1^low^ phenotype. This interpretation was further supported by our functional assays, which demonstrated significantly reduced production of IL-5, IL-6, IL-9, IL-10, and IL-13 by melatonin-treated ILC2s.

Metabolically, we observed major shifts in the way in which glucose was handled by melatonin-treated ILC2s compared to vehicle-controls. We found that glycolysis, the TCA cycle, and oxidative phosphorylation pathways were all significantly suppressed in our RNA-seq dataset. Despite this, we found that functional glucose uptake by melatonin-treated ILC2s was not affected by melatonin treatment. We did however find increased expression of genes leading into the pentose phosphate pathway (PPP). The PPP shunts glucose away from ATP producing processes and towards NADPH producing pathways. The NADPH produced via the PPP is required for the production of glutathione (GSH) (22, 23). In melatonin-treated ILC2s we found increased expression of NRF2-regulated genes that form part of the glutathione biosynthesis pathway (53, 54). Our *in vitro* GSH detection assays also revealed an accumulation of GSH in melatonin treated cells that was not found in vehicle controls. GSH biosynthesis, as well as relying on NADPH, relies on the amino acid cysteine (24, 68). By inhibiting cysteine importation, we were able to interrupt melatonin-induced GSH accumulation and found that in doing so we also suppressed the anti-inflammatory effects of melatonin treatment. This suggested that GSH was playing a key role in the suppression of ILC2 effector functions.

We tested the translational relevance of our findings in mice by treating activated human ILC2s with melatonin and IKE. We found that melatonin increased GSH levels in human ILC2s whilst concurrently reducing their effector function. This finding was not found in cells treated with IKE. Physiologically, patients with asthma frequently experience disruptions in their melatonin levels, namely a delayed acrophase in peak serum concentrations, as well as in the amount of GSH produced in the lung (15, 21). We propose that there exists a pathophysiological link between these two phenomena, whereby disturbed melatonin production results in suppressed levels of GSH in the lung of these patients. In evaluating the translational potential of these findings, it is important to contextualize the pharmacological doses utilized in our experimental models.

In clinical practice, melatonin is often prescribed at a dose of 2mg for the average (70kg) human adult (69). However, this particular dose is indicated for sleep-related pathologies such as jet-lag and works by targeting the central nervous system where canonical melatonin receptors are abundantly expressed (69). Employing melatonin as an immunomodulatory agent therefore may require higher dosages to be effective, for example in murine models, a dose of 10mg/kg is widely used to investigate the anti-inflammatory and tissue-protective actions of melatonin (18, 29–31, 70). Moreover, local intracellular accumulation and receptor-independent actions may require higher pharmacological concentrations of melatonin to engage redox-sensitive pathways such as NRF2 compared with approaches which target the central nervous system. In our experiments with DMF, we showed that the NRF2-GSH axis could be potentially targeted independently of melatonin. This approach would capitalize both on our understanding of circadian pathophysiological pathways in asthma, and existing redox-targeted therapeutics, whilst minimizing off-target effects associated with hormone based therapies such as melatonin. Further work is needed to assess the most effective dosing regimen to target the NRF2-GSH axis in the context of allergic asthma. For example, this should include time-of-day chronotherapeutic studies that examine whether exogenous melatonin is most beneficial to be given to patients in relation to endogenous rhythms. Moreover, glutathione itself is known to also be produced rhythmically and so the same logic applies to any NRF2 activator when viewed as a therapeutic in this disease context.

In summary, our findings identify melatonin as a regulator of ILC2 effector function through a glutathione-dependent, receptor-independent pathway. Melatonin redirects cellular metabolism from glycolysis and oxidative phosphorylation towards the NADPH-generating pentose phosphate pathway, enhancing NRF2 activity and glutathione biosynthesis. These results reveal a mechanistic link between circadian hormonal rhythms, cellular redox homeostasis, and innate lymphoid cell activity. Future studies using melatonin-competent mice, genetic validation of receptor independence, and time-of-day dosing paradigms will be critical to determine whether the melatonin–glutathione axis can be exploited therapeutically to treat asthma.

## Materials and methods

### Animals

Wild type (WT) BALB/cJ, recombination-activating gene 2–deficient [Rag2^–/–^, C.B6(Cg)-Rag2tm1.1Cgn/J, RRID: IMSR_JAX:008448], and Rag2-deficient γ chain–deficient (Rag2^–/–^GC^–/–^, C;129S4-Rag2tm1.1FlvIl2rgtm1.1Flv/J, RRID: IMSR_JAX:014593) mice were obtained from The Jackson Laboratory. Female mice, aged 6 to 8 weeks, from these backgrounds were used in all experiments. Animals were housed and bred in a pathogen-free animal facility at the Keck School of Medicine, University of Southern California (USC), in accordance with protocols approved by the Institutional Animal Care and Use Committee.

### Pulmonary ILC2 isolation and ex vivo experiments

Mice were intranasally challenged with 0.5 μg of rmIL-33 (BioLegend) once per day for three days before tissue collection. On the fourth day, the mice were euthanized, lungs were dissected and digested to produce single-cell suspensions as previously described. Pulmonary ILC2s were FACS-sorted to a purity > 95% on a FACSARIA III cell sorter. ILC2s were identified as follows: live cells, CD45^+^, lineage-negative (CD3ϵ, CD4, CD5, TCRβ, TCRγδ, CD45R/B220, CD335, CD11c, CD11b, Gr-1, FcϵRIα, and Ter119), ST2^+^, and CD127^+^. Isolated ILC2s were subsequently cultured ex vivo (1x10^4^ ILC2s per well in a 96-U bottom well plate) according to established protocols (48, 49, 71–73) and treated with the following compounds: Melatonin (250µM; Sigma), DMSO (Sigma), IKE (0.25µM; MedChemExpress), dimethyl fumarate (DMF) (50µM; Sigma). Ramelteon (1µM; MedChemExpress), 4-P-PDOT (1µM; MedChemExpress), Tasimelteon (1µM; MedChemExpress), S26131 (100nM; MedChemExpress). For experiments containing morpholinos the following compounds were used, all obtained from GeneTools LLC: anti-NRF2 morpholino (5µM; GCGGTGGCAACTCCAAGTCCATCAT), Control morpholino (5µM; CCTCTTACCTACCTCAGTTACAATTTATATA). Concentrations used for all compounds were tested for toxicity and effectiveness and chosen based on these results and doses were selected based on prior studies demonstrating immunomodulatory effects *in vitro* and *in vivo* without toxicity.

### Models of allergic airways disease

To induce allergic airway inflammation mice were intranasally challenged with rmIL-33 (0.5ug, Biolegend), *A. alternata* (25ug, Greer Laboratories), or PBS under anesthesia. In experiments using melatonin, either melatonin (10mg/kg, Sigma) or an equivalent volume of DMSO (Sigma) was intraperitoneally injected each day for three days at 17:00, immediately after intranasal challenges had been performed. Luzindole (30mg/kg; Tocris Bioscience) was intraperitoneally injected contemporaneously with melatonin in experiments where it was used. For adoptive transfer, 8x10^4^ ILC2s were intravenously injected into *Rag2^–/–^GC^–/–^* mice, and AHR was induced as previously described (48). AHR assessment using the FinePointe RC system (Buxco Research Systems), pulmonary ILC2 and BAL fluid cell analysis, cytokine measurement in BAL supernatant, and lung histological examination were performed according to the established protocols (46, 48, 71, 74).

### Flow cytometry

FITC Mouse Lineage Cocktail (47, 48): CD3ϵ (145-2C11, Biolegend), CD4 (GK1.5, Biolegend), CD5 (53-7.3, Biolegend), TCRβ (H57-597, Biolegend), TCRγδ (UC7-13D5, Biolegend), B220/CD45R (RA3-6B2, Biolegend), Gr-1 (RB6-8C5, Biolegend), CD11c (N418, Biolegend), CD11b (M1/70, Biolegend), Ter119 (TER-119, Biolegend), FcϵRIa (MAR-1, Biolegend), CD335 (29A1.4, Biolegend). PE-Cy7 anti-mouse CD127 (A7R34, Biolegend), APC-Cy7 anti-mouse CD45 (30-F11, Biolegend), PerCP-eFluor 710 anti-mouse IL-33R/ST2 (RMST2-2, Biolegend). FITC anti-human lineage cocktail (47, 48): CD3 (UCHT1, Biolegend), CD14 (HCD14, Biolegend), CD16 (3G8, Biolegend), CD19 (HIB19, Biolegend), CD20 (2H7, Biolegend), CD56 (HCD56, Biolegend) CD235a (HI264, Biolegend), FCϵRIa (AER-37, Biolegend), CD1a (HI149, Biolegend), CD123 (6H6, Biolegend) and CD5 (L17F12, Biolegend). APCCy7 CD45 (HI30, Biolegend), PECy7 CD127 (A019D5, Biolegend) and PE CRTH2 (BM16, Biolegend). Post-isolation and treatment with melatonin the following antibodies were used to quantify expression of ILC2 cell surface markers: APC KLRG1(2F1; 1:100; Biologend) and PECy7 GLUT1(NB110-39113; 1:100; Novus). Immunophenotyping of murine BALF samples was performed using the following markers: Live/Dead fixable aqua (1:1000; Thermofisher), PE-Cy7 CD45 (1:100; Biolegend), APC LY6G (1:100; Biolegend), eF450 CD11b (1:100; Invitrogen), APC Cy7 CD11c (1:100; Biolegend), PE Siglec-F (1:100; BD Biosciences). For intranuclear staining, ILC2s were fixed and permeabilized using the Foxp3 Transcription Factor Staining Kit (Thermo Fisher Scientific) according to the manufacturer’s instructions and PE GATA-3 (TWAJ; 1:200; Invitrogen) was used. For targets with no existing fluorophore conjugated antibody the following primary antibodies were used: anti NRF2 polyclonal (1:200, Thermofisher, PA5-27882). GCLC polyclonal (1:200, Proteintech, 12601-1-AP). GCLM polyclonal (1:200, Proteintech, 14241-1-AP), GLRX polyclonal (1:200, Proteintech, 15804-1-AP). Secondary antibodies were then used for detection as follows: AlexaFluor488 goat anti-rabbit (1:500, Invitrogen, A11008), AlexaFluor647 goat anti-rabbit (1:500, Invitrogen, A21244). For intracellular staining the BD Cytofix/Cytoperm kit was used according to the manufacturer’s instructions and PE anti-mouse IL-13 (eBio13A; 1:200; Thermo Fisher Scientific) and APC anti-mouse/human IL-5 (TRFK5; 1:200; BioLegend) were used.

Cell sorting was performed using a BD FACSAria III cell sorter, whilst flow cytometric analysis was performed on a BD FACSCanto II. Data was acquired using FACSDiva software and subsequently analyzed in FlowJo v10.10.0.

### Human studies

Human ILC2s were obtained from peripheral blood samples of five healthy anonymous adult volunteers, obtained following written consent. All participants were clinically well. Experimental protocols were approved by the USC Institutional Review Board and conducted in accordance with the principles of the Declaration of Helsinki.

### Human ILC2 isolation and culture

Human peripheral blood ILC2s were isolated from total PBMCs from a total of five individual donors to a purity of >95% on a FACSARIA III system. Blood was first diluted 1:1 in PBS, and PBMCs were isolated using SepMate-50 separation tubes (STEMCELL Technologies) according to the manufacturer’s instructions. RBC lysis (BioLegend) was performed and CRTH2^+^ cells were then isolated using the CRTH2 MicroBead Kit (Myltenyi Biotec) according to the manufacturer’s instructions. Human ILC2s were identified as CD45^+^, Lineage^−^ (CD3, CD5, CD14, CD16, CD19, CD20, CD56, CD235a, CD1a, CD123), CD127^+^, and CRTH2^+^. Isolated ILC2s were cultured at 37 °C (2 × 10^4^/ml) for 72 h in cRPMi supplemented with rhIL-2 (20 ng/ml; BioLegend), rhIL-7 (20 ng/ml; BioLegend), and rhIL-33 (100 ng/ml; BioLegend) in U-bottom 96-well plates. When indicated, 250µM melatonin and 0.25 µM IKE or corresponding DMSO control was added to cultures for the indicated readouts.

### Cytokine quantification

Cytokine concentrations in culture supernatants and BALF samples were quantified using either LEGENDplex Mouse Th Panel or LEGENDplex Human Th2 Panel (BioLegend) per the manufacturer’s instructions and described previously (47, 71, 73, 75, 76).

### Bioenergetic profiling

The real-time OCR was measured using a Seahorse Mini HS XF instrument (Agilent). Following the indicated experimental design, 5 × 10^4^ FACS-sorted ILC2s were plated on a Seahorse XFp PDL-coated cell culture miniplate in triplicates in FBS/Phenol red free Seahorse media supplemented with 1 mM pyruvate, 2 mM glutamine, and 10 mM glucose. A T cell metabolic profiling assay was then performed (Agilent). Briefly following baseline measurements, 1.5 µM oligomycin, 2.5 µM BAM15, and 0.5 µM rotenone/antimycin A were sequentially injected in the culture, and oxygen levels were measured in triplicates following each injection.

### Metabolic assays

Isolated lung activated ILC2s were cultured in RPMI 1640 with 5% FBS, 2 mM L-glutamine, 100 U/ml Pen-Strep in addition to rmIL-2 (10ng/mL) and rmIL-7 (10ng/mL) at 37 °C. Cells were treated with melatonin (250 µM; Sigma) or an equivalent volume of DMSO (Sigma). After 24 hours cells were homogenized in cold PBS. Samples were subsequently analyzed for Glucose-6-phosphate and Fructose-6-phosphate levels following the manufacturer’s instructions respectively from MAK014 and MAK020 Assay Kits (Sigma). L-lactate levels were analyzed following the Glycolysis Cell-Based Assay Kit (#600450, Cayman Chemicals). Cells were lysed in NP40 buffer and protein was quantified using Pierce BCA Protein Assay Kit (Sigma). L-lactate levels were normalized to protein levels for each well individually. Intracellular glutathione was detected and quantified using the Intracellular glutathione (GSH) Detection Assay Kit (ab112132; Abcam) according to the manufacturer’s instructions. For NADPH and β-hydroxybutyrate quantification 1x10^6^ ILC2s were cultured per well as previously described. NADPH was then quantified via colorimetric assay (WST-8; MedChemExpress) from cell lysates following the manufacturer’s instructions. β-Hydroxybutyrate was quantified using the colorimetric beta Hydroxybutyrate (beta HB) Assay Kit (ab83390; Abcam) according to the manufacturer’s specifications for both culture supernatant and cell lysates. To assess mitochondrial morphology and functional states MitoTracker Green FM Dye, MitoTracker Red FM Dye, and TMRM, (all from Thermo Fisher Scientific) were used according to the manufacturer instructions. Glucose uptake assays were performed using 2-NBDG as previously described (45).

### Histology

Lungs were collected and stored in 10% neutral buffered formalin. Tissues were then embedded in paraffin, and sections of 4µm were prepared for hematoxylin and eosin staining. Histology pictures were acquired on a KeyenceBZ-9000 microscope (Keyence). Epithelial thickness was analyzed with the QuPath analysis software (version 0.5.1). Inflammation grading was carried out independently by 3 researchers according to established protocols (77) and to the following scale: 0= No inflammation, 1= small pockets of cellular infiltrations, 2= small pockets of infiltrations less than 3 cells deep surrounding more than one airway or blood vessel, 3= infiltrations more than 3 cells deep found surrounding more than one but less than 50% of the total airways, 4=most airways surrounded by significant immune cell infiltrations, 5= the majority of airways are surrounded by significant immune cell infiltrations which also are seen present in alveolar beds.

### RNA-seq and data analysis

Activated murine ILC2s were cultured for 24h ± Melatonin (250µM)/DMSO, lysed in RLT buffer (Qiagen), and RNA was extracted using the MicroRNeasy kit (Qiagen). For each sample, a total of 10 pg of RNA was used to generate cDNA (SMARTer Ultra Low Input RNA v3 kit, Clontech) for library preparation. Samples were then amplified and sequenced on a NextSeq 500 system (Illumina), where an average of 30 million reads were generated from each sample. Raw reads were further processed on Partek Flow software, version 12.9.0 (Partek Inc). Raw reads were trimmed by quality score (min Phred score 35 from both ends and trimmed reads shorter than 25 bp were discarded). Trimmed reads were aligned by STAR 2.7.8.a with mouse reference index mm39. Aligned reads were further quantified to Gencode M33 with Partek E/M algorithm. Genes with <10 counts in all samples were removed from the analysis. The remaining genes were subjected to differential expression analysis by DESeq2 with median ratio normalization. Pathway analyses (KEGG and WikiPathways) were conducted on the ShinyGo 0.80 platform (78), using an FDR cut off of 0.05 and a minimum pathway size of 2. Meta-analyses of publicly available single cell RNAseq datasets were performed using CZ CELLxGENE Discover (79).

### Statistical analysis

Data are presented as mean ± SEM and analyzed using GraphPad Prism software (version 10.5.0). A two-tailed Student’s t test for unpaired or paired data was applied for comparisons between two groups, except for multigroup comparisons where one-way or two-way ANOVAs were used as appropriate with *post-hoc* Tukey’s t-tests. Throughout, the outcomes of statistical testing are shown as: ns=P>0.05 *P< 0.05, **P< 0.01, ***P< 0.001, ****P< 0.0001.

## Data Availability

The bulk RNA-seq data have been uploaded to the Gene Expression Omnibus database (GSE327373). Additional information necessary to facilitate the reanalysis of the data featured in this paper will be made available by the lead contact upon reasonable request.

## Supplementary material

Supplementary Figure 1 Absolute BALF cell counts from allergen and cytokine challenged mice. **(A–D)** total BALF eosinophils and neutrophils were quantified in samples taken from Alternaria-exposed mice **(A, B)**, and IL-33 challenged mice **(C, D)**. Data are mean ± SEM; n = 5, analyzed by one-way ANOVA with *post-hoc* Tukey’s t-tests.

Supplementary Figure 2 Gating strategy for the identification of ILC2. Representative FACS plots showing how ILC2s were identified by flow cytometry. Following initial size gating, doublet exclusion and live criteria, cells were classified as CD45+, Lineage-, CD127+, ST2 +.

Supplementary Figure 3 Transcriptomic meta-analyses of melatonin receptor and antioxidant pathway expression. **(A)** Aggregated results of 49 murine scRNAseq datasets showing expression of genes for canonical melatonin receptors, melatonin synthesis, and the NRF2-Glutathione pathway. **(B)** Aggregated results of 295 human scRNAseq datasets showing expression of genes for canonical melatonin receptors, melatonin synthesis, and the NRF2-Glutathione pathway. **(C)** Human lung ILC2 expression of genes for canonical melatonin receptors, melatonin synthesis, and the NRF2-Glutathione pathway aggregated from 2 independent scRNAseq datasets deposited in CZ CELLxGENE Discover. **(D)** Murine ILC2s were only detected in the dataset described in Gu et al. (2024), the gene expression results of which are presented here.

Melatonin suppresses ILC2 activity ex-vivo. **(A)** Overview of experimental procedure. **(B-F)** After 24h in culture with either 250uM melatonin or vehicle control the concentrations of IL-5 (B), IL-13 **(C)**, IL-9 **(D)** IL-10 **(E)**, and IL-6 **(F)** was determined by LEGENDPlex assay. n= 10 per treatment group. **(G)** KLRG1 expression levels on melatonin and vehicle treated ILC2s, n=3. **(H)** intracellular IL-5 staining in melatonin and vehicle treated ILC2s, n=3. **(I)** intracellular IL-13 staining in melatonin and vehicle treated ILC2s, n=3. **(J)** The expression of GATA3 transcription factor was quantified by flow cytometry in melatonin and vehicle treated ILC2s, n=3. **(K)** heatmap showing relative expression of a ILC2-related genes between melatonin and vehicle treated cell populations. **(B–J)** Data are mean ± SEM, analyzed by two-tailed Student’s t-tests.

Supplementary Figure 5 Enrichment analysis of upregulated and downregulated pathways. **(A, B)** Top 10 upregulated gene pathways according to KEGG database **(A)** and WikiPathways **(B–D)** Top 10 downregulated gene pathways according to KEGG database **(C)** and WikiPathways **(D)**. **(A–D)** Pathway analyses were carried out using ShinyGO v.0.85.1. The size of each dot represents the number of gene overlaps in each pathway, colors indicate P value.

Supplementary Figure 6 Melatonin treatment suppresses β-oxidation pathways in ILC2s. **(A)** Heatmap showing relative gene expression changes between vehicle and melatonin treated ILC2s. **(B)** Schematic outline of the β-oxidation pathway for the breakdown of fatty acids. Arrows added in purple to show the direction of gene changes for key enzymes involved in this pathway following melatonin treatment. **(C)** β-hydroxybutyrate was quantified in the supernatant of aILC2s incubated for 24h with and without melatonin. **(D)** aILC2s were lysed post 24h incubation with and without melatonin and the quantity of β-hydroxybutyrate in each lysate was quantified. **(C, D)** Data are mean ± SEM, analyzed by two-tailed Student’s t-tests.
