# Supplementary figures and images for: Melatonin suppresses ILC2-driven airway hyperreactivity via glutathione-dependent metabolic reprogramming
Source: Front Immunol. 2026 May 11;17:1845654. doi: 10.3389/fimmu.2026.1845654 (PMC13199098; doi:10.3389/fimmu.2026.1845654)

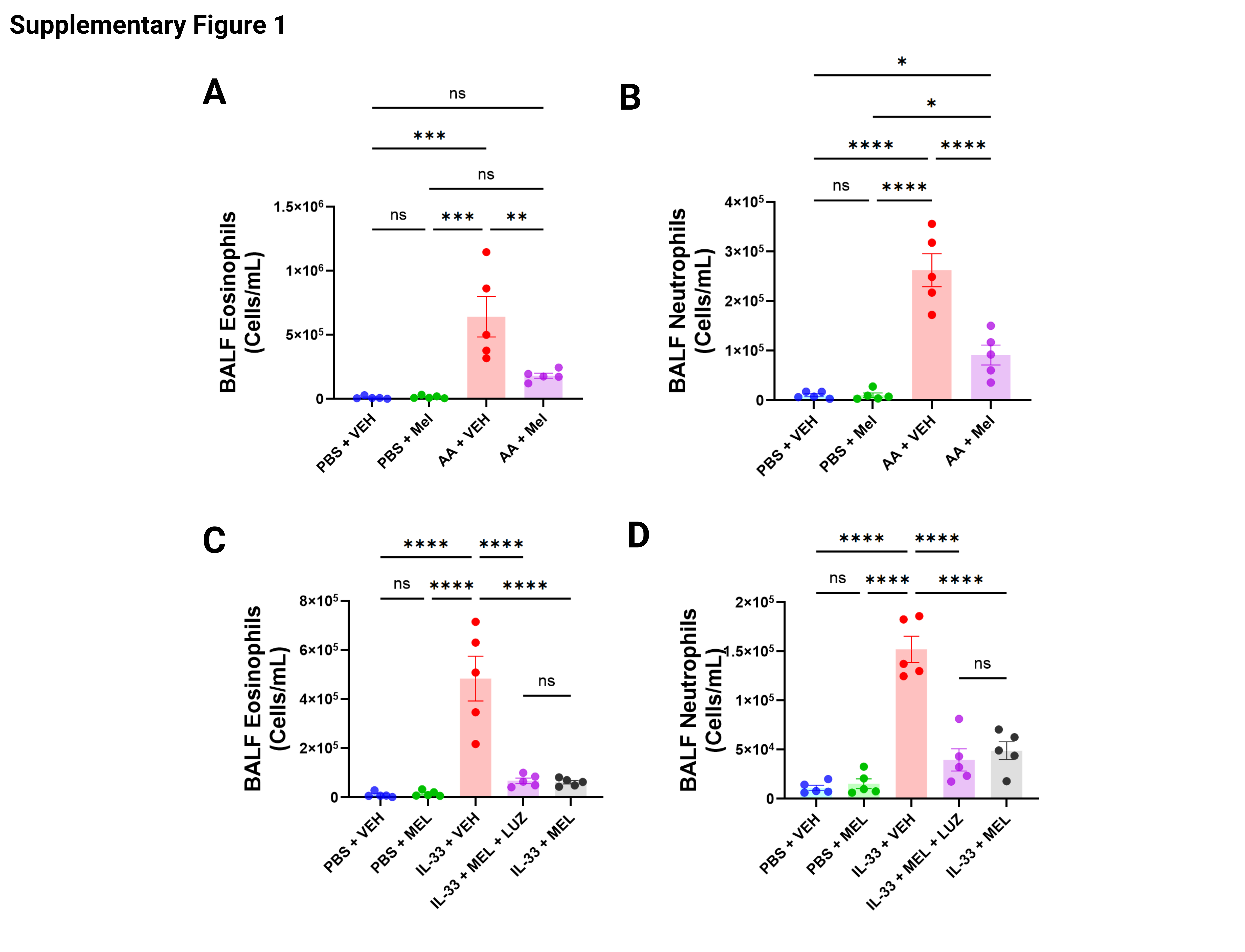

Supplement: Supplementary Figure 1 — Absolute BALF cell counts from allergen and cytokine challenged mice. (A–D) total BALF eosinophils and neutrophils were quantified in samples taken from Alternaria-exposed mice (A, B), and IL-33 challenged mice (C, D). Data are mean ± SEM; n = 5, analyzed by one-way ANOVA with post-hoc Tukey’s t-tests. [file Image1.jpeg]

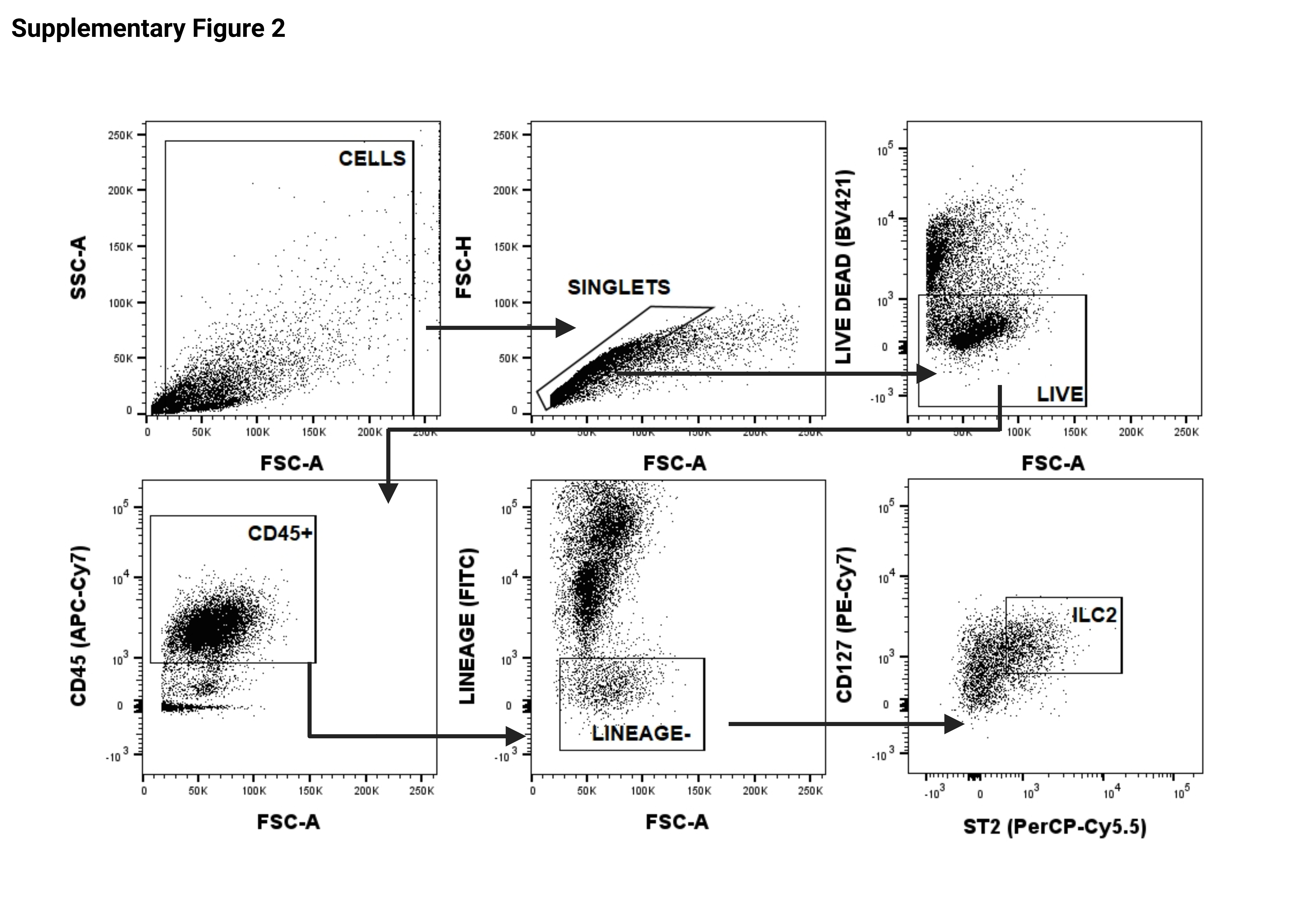

Supplement: Supplementary Figure 2 — Gating strategy for the identification of ILC2. Representative FACS plots showing how ILC2s were identified by flow cytometry. Following initial size gating, doublet exclusion and live criteria, cells were classified as CD45+, Lineage-, CD127+, ST2 +. [file Image2.jpeg]

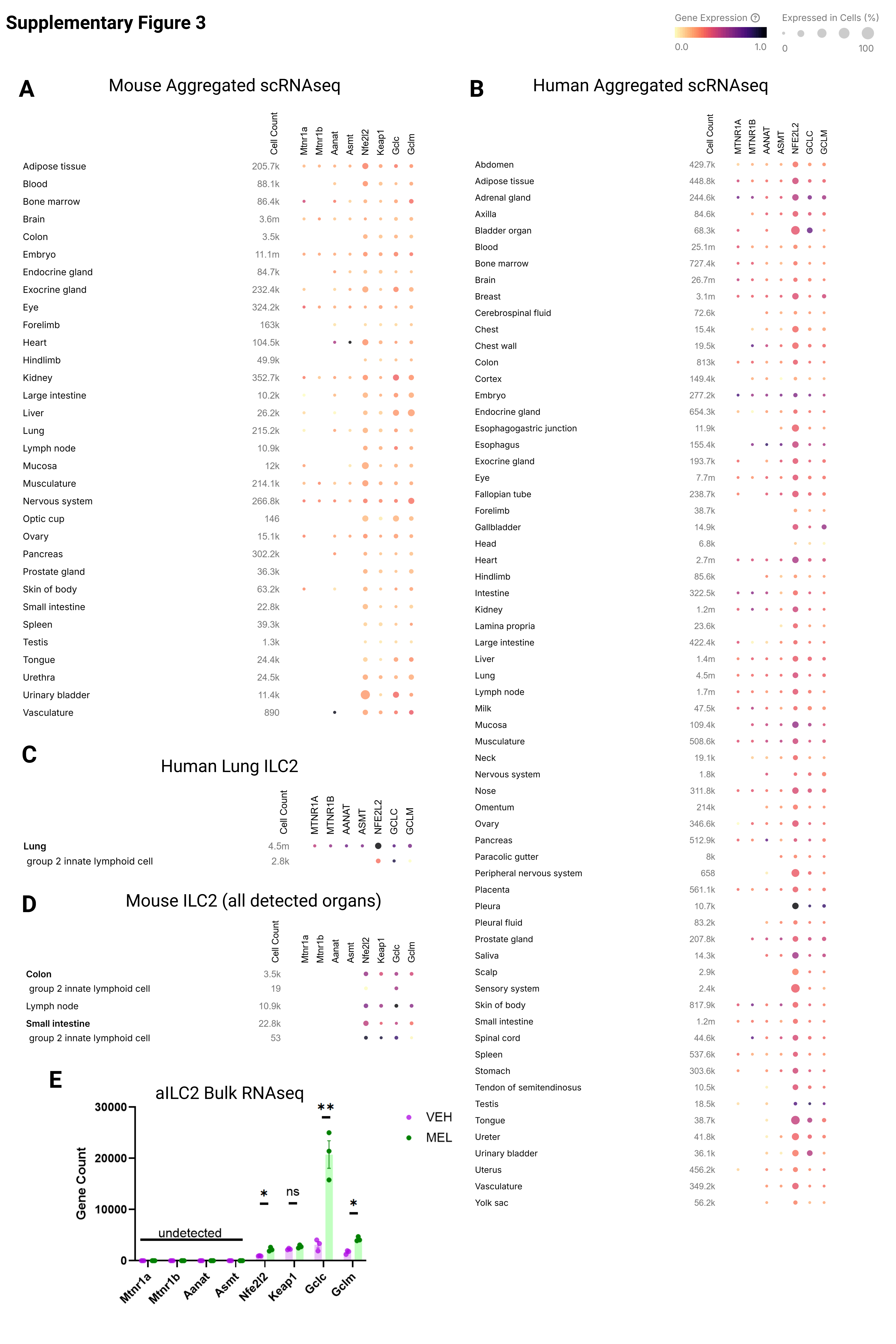

Supplement: Supplementary Figure 3 — Transcriptomic meta-analyses of melatonin receptor and antioxidant pathway expression. (A) Aggregated results of 49 murine scRNAseq datasets showing expression of genes for canonical melatonin receptors, melatonin synthesis, and the NRF2-Glutathione pathway. (B) Aggregated results of 295 human scRNAseq datasets showing expression of genes for canonical melatonin receptors, melatonin synthesis, and the NRF2-Glutathione pathway. (C) Human lung ILC2 expression of genes for canonical melatonin receptors, melatonin synthesis, and the NRF2-Glutathione pathway aggregated from 2 independent scRNAseq datasets deposited in CZ CELLxGENE Discover. (D) Murine ILC2s were only detected in the dataset described in Gu et al. (2024), the gene expression results of which are presented here. [file Image3.jpeg]

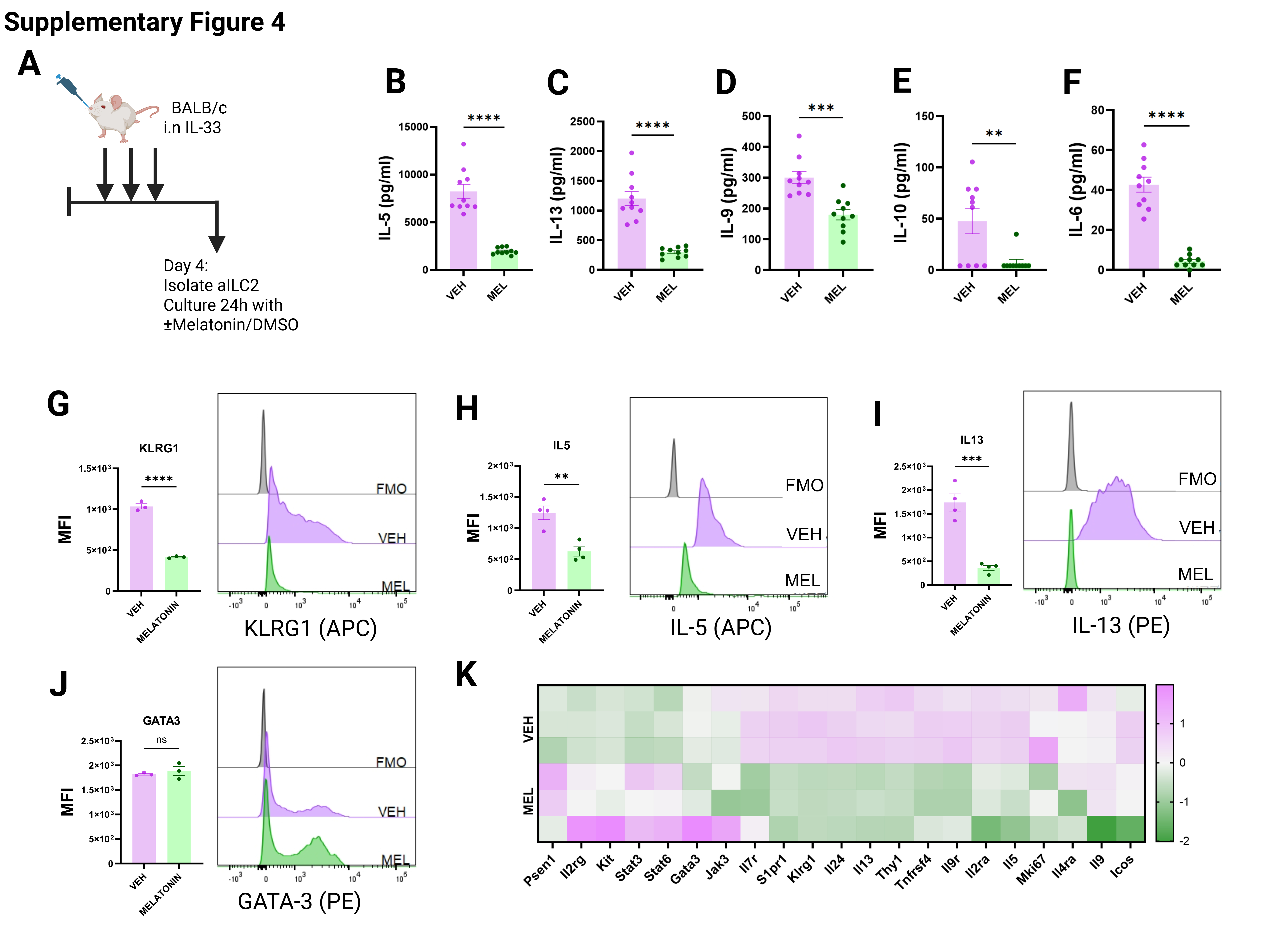

Supplement: Supplementary file 4 — Melatonin suppresses ILC2 activity ex-vivo. (A) Overview of experimental procedure. (B-F) After 24h in culture with either 250uM melatonin or vehicle control the concentrations of IL-5 (B), IL-13 (C), IL-9 (D) IL-10 (E), and IL-6 (F) was determined by LEGENDPlex assay. n= 10 per treatment group. (G) KLRG1 expression levels on melatonin and vehicle treated ILC2s, n=3. (H) intracellular IL-5 staining in melatonin and vehicle treated ILC2s, n=3. (I) intracellular IL-13 staining in melatonin and vehicle treated ILC2s, n=3. (J) The expression of GATA3 transcription factor was quantified by flow cytometry in melatonin and vehicle treated ILC2s, n=3. (K) heatmap showing relative expression of a ILC2-related genes between melatonin and vehicle treated cell populations. (B–J) Data are mean ± SEM, analyzed by two-tailed Student’s t-tests. [file Image4.jpeg]

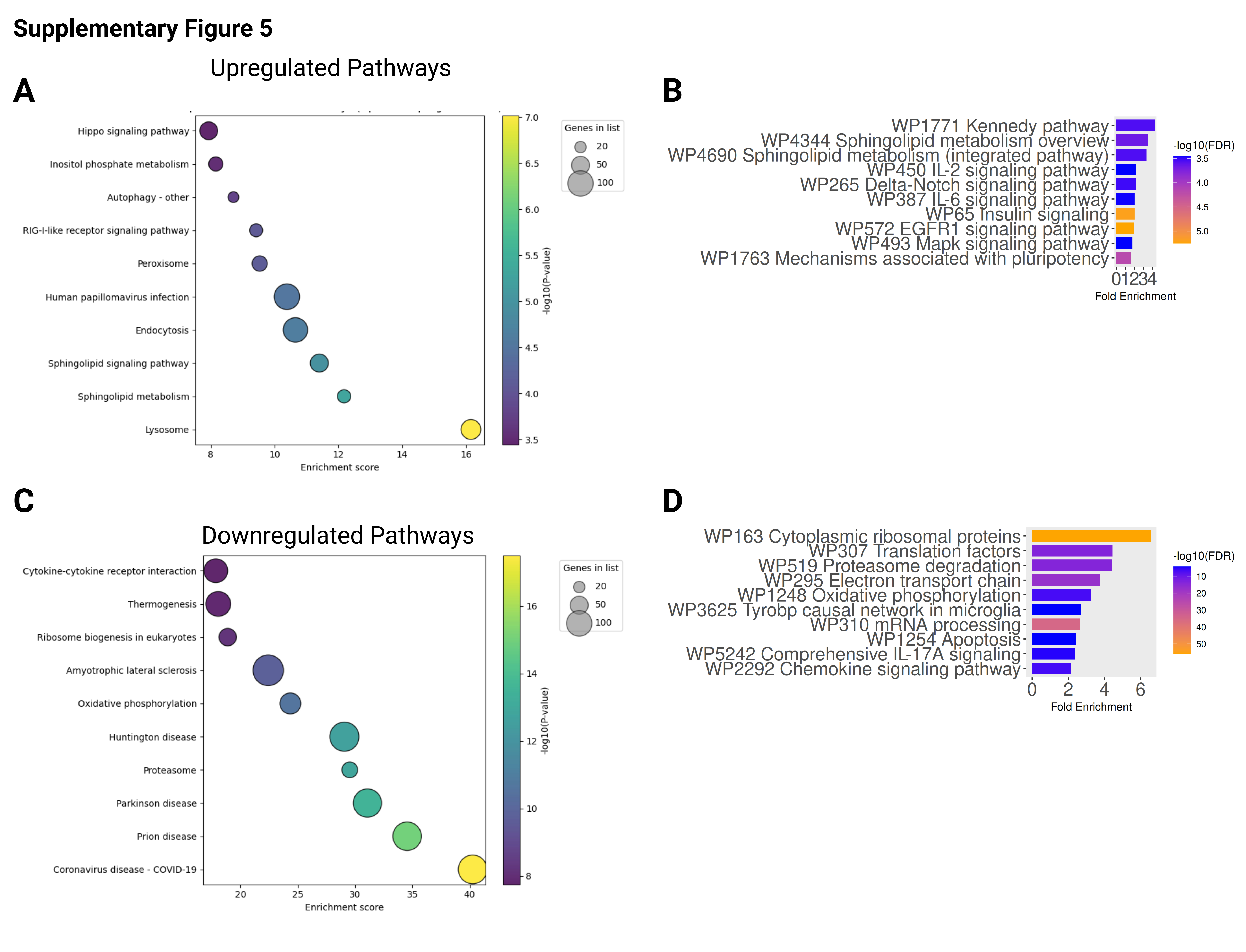

Supplement: Supplementary Figure 5 — Enrichment analysis of upregulated and downregulated pathways. (A, B) Top 10 upregulated gene pathways according to KEGG database (A) and WikiPathways (B–D) Top 10 downregulated gene pathways according to KEGG database (C) and WikiPathways (D). (A–D) Pathway analyses were carried out using ShinyGO v.0.85.1. The size of each dot represents the number of gene overlaps in each pathway, colors indicate P value. [file Image5.jpeg]

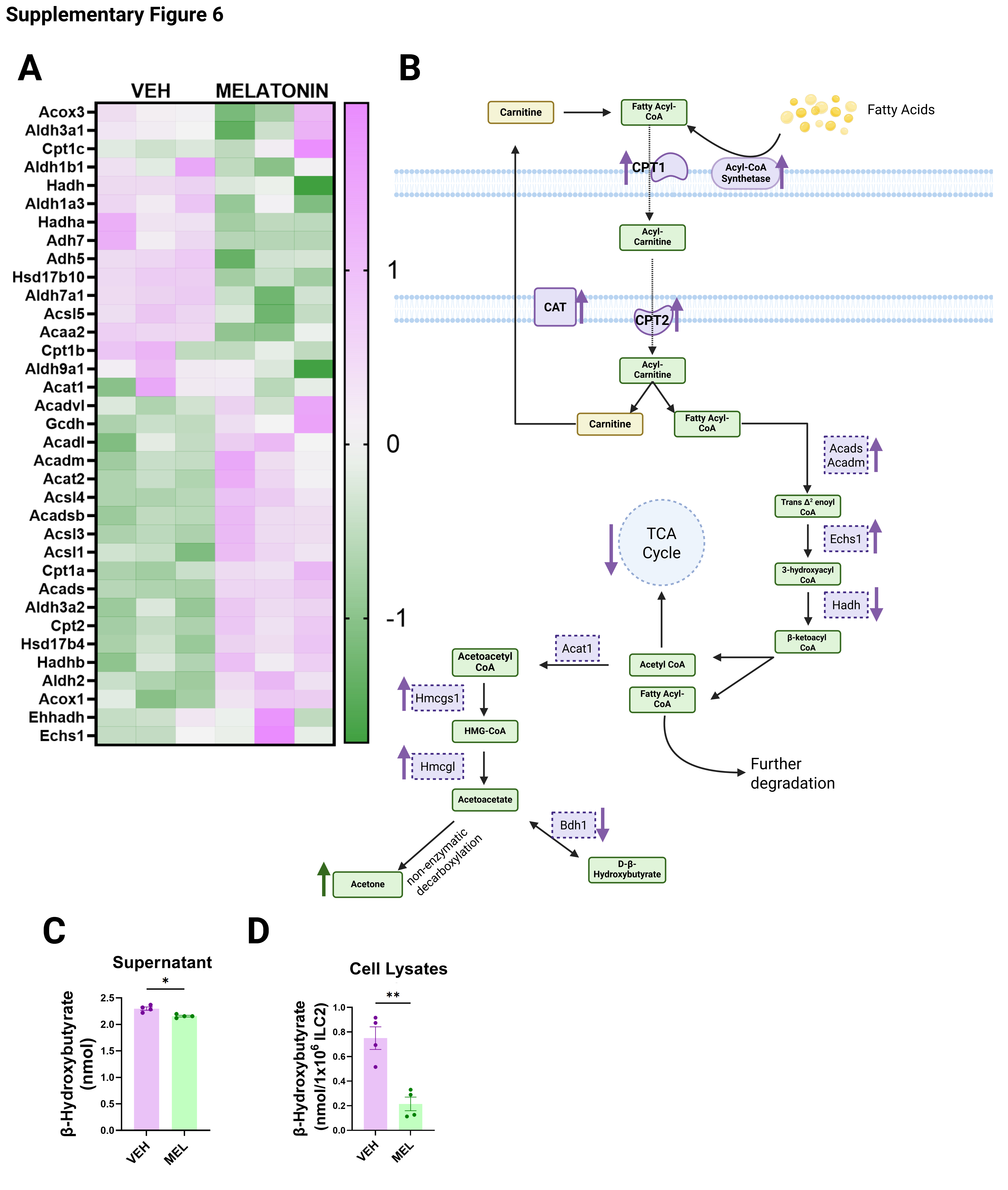

Supplement: Supplementary Figure 6 — Melatonin treatment suppresses β-oxidation pathways in ILC2s. (A) Heatmap showing relative gene expression changes between vehicle and melatonin treated ILC2s. (B) Schematic outline of the β-oxidation pathway for the breakdown of fatty acids. Arrows added in purple to show the direction of gene changes for key enzymes involved in this pathway following melatonin treatment. (C) β-hydroxybutyrate was quantified in the supernatant of aILC2s incubated for 24h with and without melatonin. (D) aILC2s were lysed post 24h incubation with and without melatonin and the quantity of β-hydroxybutyrate in each lysate was quantified. (C, D) Data are mean ± SEM, analyzed by two-tailed Student’s t-tests. [file Image6.jpeg]
